# When Is Hub Gene Selection Better than Standard Meta-Analysis?

**DOI:** 10.1371/journal.pone.0061505

**Published:** 2013-04-17

**Authors:** Peter Langfelder, Paul S. Mischel, Steve Horvath

**Affiliations:** 1 Department of Human Genetics, University of California Los Angeles, Los Angeles, California, United States of America; 2 Department of Pathology and Laboratory Medicine, University of California Los Angeles, Los Angeles, California, United States of America; 3 Departments of Human Genetics and Biostatistics, University of California Los Angeles, Los Angeles, California, United States of America; King Abdullah University of Science and Technology, Saudi Arabia

## Abstract

Since hub nodes have been found to play important roles in many networks, highly connected hub genes are expected to play an important role in biology as well. However, the empirical evidence remains ambiguous. An open question is whether (or when) hub gene selection leads to more meaningful gene lists than a standard statistical analysis based on significance testing when analyzing genomic data sets (e.g., gene expression or DNA methylation data). Here we address this question for the special case when multiple genomic data sets are available. This is of great practical importance since for many research questions multiple data sets are publicly available. In this case, the data analyst can decide between a standard statistical approach (e.g., based on meta-analysis) and a co-expression network analysis approach that selects intramodular hubs in consensus modules. We assess the performance of these two types of approaches according to two criteria. The first criterion evaluates the biological insights gained and is relevant in basic research. The second criterion evaluates the validation success (reproducibility) in independent data sets and often applies in clinical diagnostic or prognostic applications. We compare meta-analysis with consensus network analysis based on weighted correlation network analysis (WGCNA) in three comprehensive and unbiased empirical studies: (1) Finding genes predictive of lung cancer survival, (2) finding methylation markers related to age, and (3) finding mouse genes related to total cholesterol. The results demonstrate that intramodular hub gene status with respect to consensus modules is more useful than a meta-analysis p-value when identifying biologically meaningful gene lists (reflecting criterion 1). However, standard meta-analysis methods perform as good as (if not better than) a consensus network approach in terms of validation success (criterion 2). The article also reports a comparison of meta-analysis techniques applied to gene expression data and presents novel R functions for carrying out consensus network analysis, network based screening, and meta analysis.

## Introduction

Genomic data (in particular gene expression data) have been analyzed with network methods for over a decade [1]–[10]. Since highly connected hub nodes are central to the network’s architecture [9], [11]–[14] and protein knockout experiments have shown that hub proteins tend to be essential for survival in lower organisms (yeast, fly, worm) [7], [11], [12], [15], [16], many articles have explored the role of hub genes in higher organisms (including humans and mice). While there is an ongoing debate in the literature regarding the importance of hub genes, it is fair to say hubs are often *not* important. We have argued that it is critical to focus on *intramodular* hubs instead of whole network hubs when it comes to coexpression network applications [3], [6]. One can theoretically characterize network modules (clusters of interconnected nodes) whose intramodular hub genes will be significantly related to a trait (e.g. disease status, survival time, or age) [17], [18]. As expected, intramodular hubs in disease related modules are often of clinical importance, e.g. intramodular hubs in a cell proliferation module turn out to be correlated with cancer survival time in glioblastoma multiforme [6], [19]. To find biologically relevant modules and corresponding intramodular hubs, weighted correlation network analysis (WGCNA, [3], [6]) typically proceeds along the following steps. First, the input variables (e.g., thousands of gene expression profiles) are clustered to identify sets of highly interconnected nodes, referred to as modules. The rationale for this step is that clusters (modules) of co-expressed genes are often strongly enriched in specific functional categories or cell markers [2], [20]–[22]. Second, biologically relevant modules are identified using external information, e.g., by correlating the module genes with a clinical trait of interest (such as disease status, survival time, cholesterol levels). This module centric analysis alleviates the multiple testing problem inherent in high dimensional data since it focuses on the relationship between a few modules and the sample trait. Third, a measure of intramodular connectivity with respect to relevant modules is used to select intramodular hubs. The geometric interpretation of correlation network analysis can be used to argue that intramodular connectivity can be interpreted as a fuzzy measure of module membership [17], [18]. Thus, a gene screening approach that considers intramodular connectivity amounts to a pathway-based gene screening method. Empirical evidence shows that the resulting systems biological gene screening methods can lead to important biological insights [6]–[8], [10], [23]–[28]. Gene connectivity has not only been used for identifying hubs but also for identifying differentially connected genes [25], [29], [30].

Despite multiple successful case studies the use of network connectivity for gene selection (more generally for variable screening) is still debated, in part because it lacks the theoretical basis that underlies established marginal statistical and model-based gene selection procedures. Therefore, it is of great practical importance to decide whether a marginal differential expression analysis (for example, based on a Student t-test or a fold change criterion) or a co-expression network analysis should be used to find disease related genes based on gene expression data (or other high dimensional -omics data). Our previous attempts to answer this question in generality have failed since our preliminary results from theoretical and simulation studies could not be corroborated in comprehensive real data applications. Reasons why this is a hard question may include the high rates of false positives when selecting trait related genes, the non-robustness of module detection procedures, as well as non-reproducibility due to technical variation, batch effects, tissue heterogeneity etc. Here we narrow our attention to the special situation when multiple independent gene expression data are available (e.g., collected from public repositories such as Gene Expression Omnibus [31] or ArrayExpress [32]). Multiple data sets not only allow one to robustly define lists of trait-related genes but also to define consensus network modules (i.e., modules that are present in all data sets). Using 3 diverse empirical case studies plus simulations, we address the following questions when dealing with multiple genomic data sets.

1. Are whole-network hub genes relevant or should one exclusively focus on intramodular hubs? Answer: Our correlation network applications show that one should focus on intramodular hubs in trait-related modules.
2. Which standard marginal meta-analysis method (i.e., methods that ignore gene-gene relationships) results in best validation of gene/trait associations? Answer: Overall, the 9 considered methods have similar performance in our applications.
3. How to select hub genes in consensus modules? Answer: Meta-analysis techniques applied to a measure of intramodular connectivity (also known as module membership) work quite well. Just forming the average across data sets works well.
4. Do network-based gene selection strategies lead to gene lists that are biologically more informative than those based on a standard marginal approaches? Answer: Yes, gene selection based on *intramodular connectivity* leads to biologically more informative gene lists than marginal approaches in all 3 applications. In contrast, *whole-network connectivity* leads to the least informative gene lists.
5. Do network-based gene selection strategies lead to gene lists that have more reproducible trait associations than those based on a standard marginal approaches? Answer: Overall, the answer is no. Our simulations explore this further.

Thus, our findings indicate that meta-analysis of module membership (i.e., selecting intramodular hubs in consensus modules) leads to gene lists with better biological interpretability but possibly lower validation success. In other words, while network methods may be preferable when learning about biology, standard marginal meta-analysis methods may be better suited for selecting candidate biomarkers.

## Results

### Overview of Standard Meta-analysis Methods Used in this Work

In this work we focus on comparing meta-analysis of associations quantified without regard to gene-gene relationships (meta-analysis of marginal association or *marginal meta-analysis*) to meta-analysis of module membership. Here we study three variants of the inverse normal meta analysis technique first proposed by Stouffer et al [33] and two methods that make standard meta-analysis methods applicable to a broader range of statistics. Table 1 presents a brief overview of the methods used in this article. The “inverse normal” name derives from the fact that the method uses the inverse normal distribution function to turn individual input p-values into Z statistics which are then combined into a meta-analysis Z statistic whose distribution under the null hypothesis is known (Equation 2, Methods). The three variants differ by how they weigh each study. The simplest variant proposed in [33] assigns equal weight to each study, irrespective of the number of observation used in each study (Equation 3), and we call it Stouffer’s method with equal weights. Under certain assumptions one can show that the theoretically optimal weights are 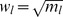 [34]–[36], where 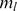 is the number of samples (more precisely, number of degrees of freedom) in each study. It should be noted that the assumptions that underlie this results are often not satisfied in real applications and hence it is meaningful to study empirically which weighting method performs best in practice. Here, in addition to the equal weight case and the theoretically optimal case 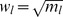 (referred to as Stouffer’s method with square root weights), we also study weights 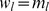 (referred to as Stouffer’s method with degree of freedom weights). Irrespective of what weights one chooses, Stouffer’s method crucially depends on the normal distribution and known variance of the input Z statistics.

**Table 1:** Overview of meta-analysis methods used in this article.

No.	Method	Variant	Input	Trafo.	Weights
1	Stouffer	equal weights	Z-statistics	None	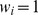
2	Stouffer	sq. root weights	Z-statistics	None	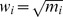
3	Stouffer	d.o.f. weights	Z-statistics	None	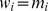
4	rankPvalue	Scale, equal weights	Var. imp.	Scale	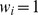
5	rankPvalue	Scale, sq. root weights	Var. imp.	Scale	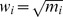
6	rankPvalue	Scale, d.o.f. weights	Var. imp.	Scale	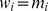
7	rankPvalue	Rank, equal weights	Var. imp.	Rank	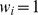
8	rankPvalue	Rank, sq. root weights	Var. imp.	Rank	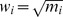
9	rankPvalue	Rank, d.o.f. weights	Var. imp.	Rank	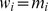

The Method and Variant columns list the names for each method that are used throughout the text and in our Figures. Var. imp. stands for a general variable importance measure; the Trafo. column indicates how the input is transformed before calculating a meta-analysis statistic; the Weights columns indicates the weights used in the calculation of the meta-analysis statistic via Equations 4 or 5.

### Meta-analysis Based on Ranking a Variable Importance Measure: RankPvalue

We consider a novel meta-analysis method, called rankPvalue, that can take as input any ordinal measure of variable importance. The rankPvalue method (and R function of the same name) relies on rankings of the variable importance measures in each input data set. A crucial assumption for the method is that the number of variables is large. This is certainly satisfied in genomic data where the number of probes is typically tens of thousands or more. Using a general variable importance measure is advantageous when it is difficult to quantify statistical significance (a p-value or Z statistic) for the input measure. Examples of such measures include network connectivity and centrality measures for which it is often difficult to define statistical significance.

There are two variants of the rankPvalue method: the *Scale* method and the *Rank* method. As indicated by its name, the *Scale* method first scales the individual importance measures in each study to mean 0 and variance 1. It then averages the statistics and relies on the central limit theorem to approximate the null distribution of the resulting meta-analysis statistic. If the assumptions of the central limit theorem are not met, then we recommend the use of the *Rank* method. As indicated by its name, the Rank method replaces the values of the importance measures by their rankings. Next the rankings are divided by the number of variables so that the resulting value lies in the unit interval. Under the null hypothesis the observed ranking of a given variable can be considered to be drawn from a uniform distribution on the unit interval. For a given variable the sum of these rankings is the meta analysis test statistic. Its distribution under the null hypothesis can be estimated from convoluting the distributions of 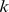 independent uniformly distributed variables. Fortunately, the convolution of uniformly distributed variables converges rapidly to the normal distribution: as few as 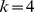 suffice [37]. A more detailed description of all meta-analysis methods is provided in Methods.

### Selecting Hub Genes in Consensus Modules: Meta-analysis of Module Membership

Since intra-modular hub genes have been shown to be biologically important in multiple previous applications, we now extend the concept of an intra-modular hub gene to multiple data sets. Our approach starts with weighted correlation network analysis (WGCNA) to identify consensus modules across the given data sets [38] (Methods). WGCNA is particularly attractive for finding consensus modules and intramodular hubs since a) one can calibrate weighted networks before combining them, b) it is straightforward to combine weighed networks across independent data sets, c) it provides module eigengenes that can be used to relate modules to sample traits (e.g. disease status), and d) it affords measures of module membership (kME), which can be used for finding hub genes in consensus modules. Consensus modules can be found using our R function *blockwiseConsensusModules* in the WGCNA R package. Hub genes in consensus modules can be found using our R function *consensusKME*. By definition, consensus modules are clusters present in all of the input data sets. We emphasize that the modules are identified in an unsupervised fashion, i.e., without regard to the clinical trait. Next, a trait-related consensus module is selected, for example, as the module with the highest *eigennode significance* (Equation 20, Methods) across the individual data sets. Finally, variables with highest overall module membership in the trait-related consensus module are identified, using a meta-analysis of module memberships (Equation 19) in the individual data sets.

### Hub Gene Selection in Consensus Modules Results in Gene Lists with Cleaner Functional Annotation

We present 3 applications that illustrate the use of meta-analysis of module membership (i.e., intra-modular hub gene selection) to study functional categories associated with a trait of interest: In Application 1, we study adenocarcinoma expression data and relate them to survival time; in Application 2 we study genome-wide blood methylation data and relate them to age; and in Application 3 we study mouse liver expression data and relate them to plasma cholesterol levels. In all 3 applications we perform a consensus module analysis (Methods) across all input data sets and identify a module associated with the trait of interest. The data used in the applications are summarized in Table 2.

**Table 2:** Overview of data sets used in this article.

Application	No.	Description	# samples	Ref.
Lung cancer	1	MSAS (Michigan)	162	[40]
	2	MSAS (HLM)	69	[40]
	3	MSAS (DFCI)	73	[40]
	4	MSAS (MSKCC)	89	[40]
	5	Bild et al	51	[41]
	6	Tomida et al	91	[42]
	7	Takeuchi et al	81	[43]
	8	Roepman et al	49	[44]
Aging	1	WB Type 1 Diabetes	190	[49]
	2	WB ovarian cancer controls	261	[50]
	3	WB healthy PMP females	87	[51]
	4	Brain frontal cortex	132	[52]
	5	Brain temporal cortex	126	[52]
	6	Brain pons areas	123	[52]
	7	Brain cerebellum	111	[52]
Mouse liver	1	CAST×B6 females	141	[27]
	2	CAST×B6 males	100	[27]
	3	B6×C3H ApoE females	134	[24]
	4	B6×C3H ApoE males	124	[24]
	5	B6×C3H wild type females	66	[55]
	6	B6×C3H wild type males	69	[55]
	7	C3H×B6 wild type females	63	[55]
	8	C3H×B6 wild type males	66	[55]
	9	Mouse Diversity Panel	196	[56]

Column # samples lists the number of samples (after our removal of potential outliers) in each data set. MSAS, Multi-Site Adenocarcinoma Study; HLM, Moffit Cancer Center; DFCI, Dana-Farber Cancer Institute; MSKCC, Memorial Sloan-Kettering Cancer Center; WB, whole blood; PMP, postmenopausal.

To compare meta-analysis of module membership to marginal meta-analysis and meta-analysis of whole-network connectivity, we use each method to select a given number 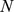 of top-ranked genes and study their enrichment in a known set of genes (the “gold standard”). As the gold standard we use Gene Ontology [39] categories or gene lists that have been strongly associated with the outcome in existing literature.

#### Genes associated with adenocarcinoma survival time in human expression data

Here we analyze 8 adenocarcinoma data sets [40]–[44] described in more detail in Methods. As gold standard for judging the biological signal in a list of survival related genes, we used enrichment with respect to the GO term “cell cycle” since cell-cycle associated genes have been observed to be among the strongest predictors of survival [45], [46] and proliferating cancer is known to be associated with a poor prognosis of survival (e.g., [47], [48]). Our results would be qualitatively the same if we choose a related term such as “cell cycle process” or “mitotic cell cycle”.

**Figure 1:**
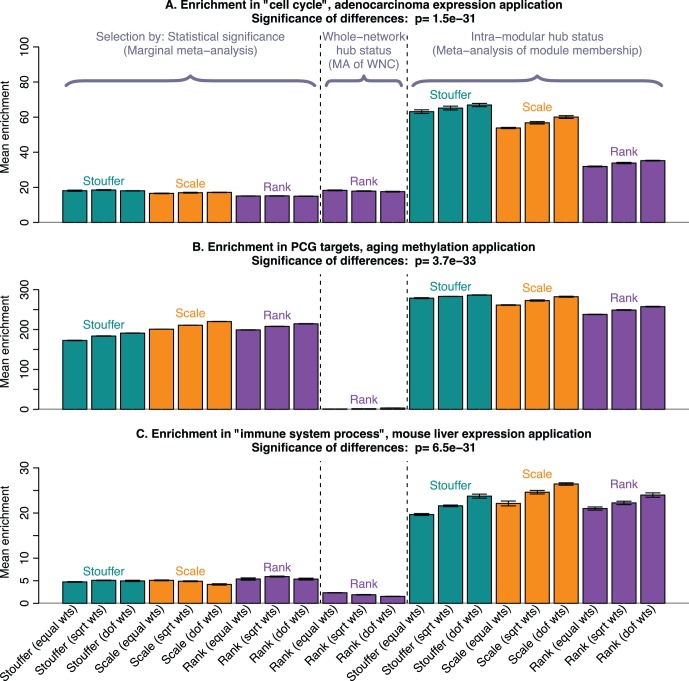
Meta-analysis of module membership leads to gene lists with stronger functional enrichment. The 3 barplots show enrichment values, defined as negative 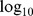 of the enrichment p-value, 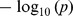, in our 3 applications. Each bar summarizes the best enrichment values obtained by the corresponding meta-analysis method. Specifically, for each method we computed the enrichment in the corresponding “gold standard” list of genes. The enrichment was calculated in the top 20, 40, 60, …, 1000 genes in the adenocarcinoma and mouse TC applications; and in 100, 200, …, 5000 genes in the aging application. The best 20% of enrichment values were retained. Each bar represents the mean of these best enrichment values, and error bars give the corresponding standard deviations. The standard deviations are not corrected for auto-correlation of enrichment values. The Kruskal-Wallis test p-value is indicated in the title. The figure shows that meta-analysis of membership in consensus modules leads to gene lists with higher enrichment and hence better biological interpretability.

The consensus module analysis (Methods and Figure S1 in Text S1 ) identified 5 modules labeled by numbers 1–5. Module 2 (93 genes) is by far most significantly associated with survival time (Figure S2 in Text S1 ). Hence, this module is a natural choice for selecting intramodular hubs related to lung cancer survival time. We emphasize that this module was selected only based on its association with survival time. The module turned out to be significantly enriched with cell cycle genes (Bonferroni-corrected hypergeometric enrichment p-value 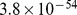, see Table S1). Figure 1A and Figure S3 (Text S1 ) report enrichment p-values (with respect to cell cycle genes) for gene lists selected by standard marginal meta-analysis, meta-analysis of module membership, and meta-analysis of whole-network connectivity, as a function of the list size. The figures show that meta-analysis of module membership (i.e., selecting intramodular hub genes in this survival time related module) results in gene lists with much stronger cell cycle gene enrichment compared to gene lists based on standard meta-analysis techniques. While intramodular hubs are clearly important, the figure also shows that meta-analysis of whole-network connectivity leads to inferior results which supports the claim that whole-network hubs are often irrelevant for important biological processes.

#### CpGs hypermethylated with age in human blood and brain methylation data

DNA methylation at the 5 position of cytosine has been observed across all vertebrate examined to date. In adult somatic tissues, DNA methylation typically occurs in a CpG dinucleotide context. It has been known for decades that age has a profound effect (both increasing and decreasing) on DNA methylation levels. Here we analyze 7 DNA methylation array data sets [49]–[52] (all measured on the Illumina Infinium HumanMethylation27 array platform) to find CpG dinucleotides that become hypermethylated with age. Most of the measured CpGs on the Illumina array are located in the promoters of genes and promoter methylation usually reduces gene expression levels.

It is well known that CpGs located in the promoters of Polycomb Group (PCG) target genes have an increased chance of becoming hypermethylated with age (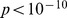) [50]. Therefore, we used enrichment with respect to PCG targets as the gold standard for judging the biological signal inherent in a list of CpGs that are positively correlated with age. The consensus module analysis identified 41 modules (Figure S4 in Text S1 ). We focused on intramodular hubs in module 6 (comprised of 517 CpGs) since its eigennode had the highest correlation with age (Figure S5 in Text S1 ). We emphasize again that the module was selected based on the correlation of the module eigengene with age, without regard to its enrichment in PCG targets. Figures 1B and S6 (Text S1 ) show enrichment p-values with respect to PCG targets for the CpG lists selected using marginal meta-analysis, meta-analysis of module membership (for selecting intramodular hub CpGs), and meta-analysis of whole-network connectivity (for selecting whole-network hubs). Selecting intramodular hub genes in the age related module (i.e., meta-analysis of module membership) leads to lists with an increased biological signal compared to marginal meta-analysis. In contrast, CpGs selected by whole-network connectivity show weak enrichment in PCG targets, illustrating the crucial distinction between whole-network hubs and intramodular hubs. While marginal meta-analysis is inferior to the meta-analysis of module membership, it nevertheless leads to highly significant enrichment p-values because in this application the biological signal is very strong.

#### Genes positively correlated with total cholesterol in mouse liver expression data

The goal of this analysis is to find genes whose expression profiles correlate positively with total cholesterol (TC) in mouse liver tissue. Since there exist no “gold standard” lists of genes related to TC, we focus on immune system genes because the immune system has been reported to have a strong connection to TC levels in mice [53], [54] Therefore, we used GO enrichment with respect to the GO term “immune system process” as the gold standard to determine which gene selection method led to the highest biological signal. We analyze 9 mouse liver gene expression data sets: 8 data sets from 4 different F2 mouse crosses [24], [27], [55] on high fat diets and a genetically more diverse Mouse Diversity Panel (MDP) [56]. Consensus module analysis identified 11 consensus modules (Figure S7 in Text S1 ). Several of the modules relate strongly to TC (Figure S8 in Text S1 ). We focus on module 2 because its eigengene is most strongly correlated with TC. Figure 1C and Figure S9 (Text S1 ) show how the enrichment (with respect to immune system process) depends on the gene selection method and the list size.

Selecting intramodular hubs (i.e., meta-analysis of module membership with respect to module 2) leads to lists of genes with more significant enrichment than marginal meta-analysis which supports the claim that studying these hub genes leads to increased biological signal. Note that the enrichment results for intramodular hubs are much more significant than those involving whole-network hubs which illustrates again that it is crucial to focus on intramodular hubs with respect to a relevant module.

### Standard Meta-analysis Methods often Lead to Better Validation Success

**Figure 2:**
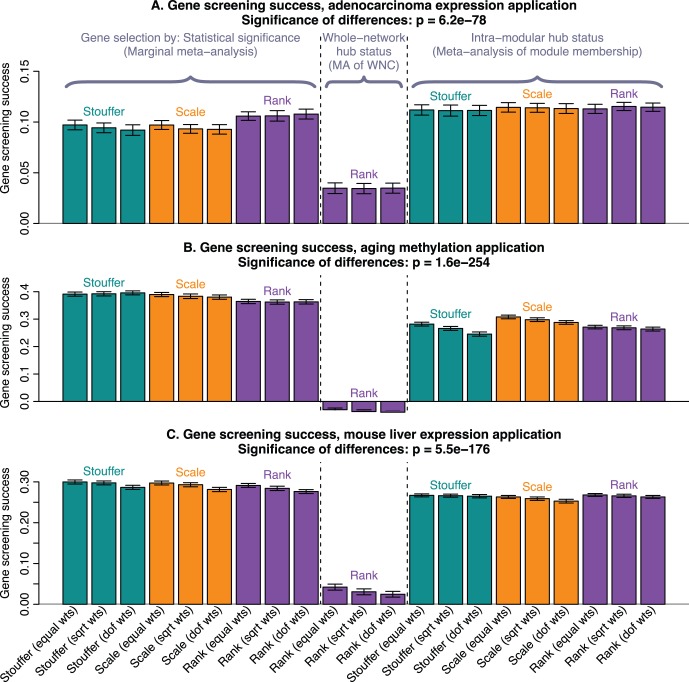
Marginal meta-analysis tends to lead to gene lists with better validation in independent data. The 3 barplots show validation success in our 3 applications. Each bar summarizes the gene screening success of the corresponding meta-analysis method. Specifically, we rank the genes using each meta-analysis method and retain the top 100 genes. We define gene screening success as the average correlation of these top 100 genes with the trait of interest in an independent validation data set, averaged over the validation sets in each application. Each bar represents the gene screening success; error bars give the corresponding standard deviation of the observed gene–trait correlations in the top 100 genes. This figure shows that, overall, marginal meta-analysis leads to gene lists with better validation success (i.e., higher correlation with the trait of interest in validation data). Adenocarcinoma expression data (panel A) present an exception in that meta-analysis of module membership results in gene lists with somewhat better validation.

We now turn our attention to the task of selecting biomarkers for a clinical trait of interest (e.g., cancer survival time, age, or total cholesterol). In this situation the primary criterion is the utility of the marker to predict the clinical trait; the biological insights gained (e.g., based on gene ontology enrichment analysis) play only a secondary role. Thus, we judge the performance of different gene selection methods by their ability to lead to lists of genes whose association with the clinical trait is preserved (reproducible) in independent data sets. Since each of our applications involves multiple independent data sets, we are able to select one of these data sets as validation set while the remaining data sets are “training” (or discovery) data for selecting lists of potential biomarkers. Thus, given a total of 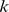 independent data sets, 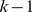 data sets are used to select the biomarkers (e.g., based on standard meta-analysis or consensus module based analysis) and the last data set is used as a validation data set to measure validation success of the different gene lists. To avoid biasing the results, we applied the consensus module analysis only to the 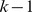 training data sets and selected intramodular hubs with respect to these training data. The validation success of a gene list (and corresponding variable selection method) is defined by the average correlation of the selected genes with the trait of interest (survival time deviance, age, and total cholesterol) in the validation data set. Our results are largely unchanged if other measures of validation success are chosen. By cycling through the 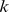 different possible choices of validation data sets, we arrived at 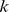 corresponding estimates of validation success that can be summarized using the mean value (see Figure 2).

As expected, prioritizing variables (genes) according to whole-network connectivity leads to gene lists with poor validation success in all 3 applications. This confirms what statisticians already know: whole-network connectivity is of little value for variable selection. We hypothesized that standard meta-analysis would also outperform intramodular hub gene selection since a strong marginal association is a critical characteristic of a trait-related biomarker. This hypothesis is confirmed in 2 of the 3 applications: When finding biomarkers for age in human DNA methylation data sets, and (somewhat less so) biomarkers of total cholesterol in mouse liver expression data, marginal meta-analysis leads to increased validation success compared to selecting intramodular hub genes in consensus modules. This is illustrated in Figures 2B and 2C. Surprisingly, the hypothesis was proven wrong for adenocarcinoma survival time. Here selecting intramodular hubs in the consensus module related to survival time leads to better validation success than marginal meta-analysis (Figure 2A). A detailed analysis of screening success as a function of the number of selected genes (Figure S10 in Text S1 ) confirms that, in this application, selecting intramodular hub genes is superior. To understand under what circumstances intramodular hub selection can be superior to marginal meta-analysis, we noted that the signal in the adenocarcinoma data is very weak: While the average validation success in the aging and mouse TC applications is around 0.4 and 0.3 (Figures 2B and 2C), the average validation success in the adenocarcinoma application is only around 0.12 (Figure 2A). Several factors likely contribute to the low signal, for example the high heterogeneity across adenocarcinoma biopsy samples as well as the fact that the data were measured on various different Affymetrix and Agilent platforms. Since hub gene selection bested marginal meta-analysis only in the application with weak signal, we hypothesized that selecting biomarkers on the basis of consensus module membership could have some merit when dealing with a weak signal. To explore this further, we carried out simulation studies described in the following.

### Simulation Studies

To better understand why meta-analysis of module membership can sometimes (e.g., in our adenocarcinoma application) lead to superior candidate biomarker lists, we performed a simulation study. Using the gene expression simulation functions in the WGCNA R package, we simulated 8 data sets with the same module structure that consists of 10 modules. One of the large modules (labeled 1) contains 3 small submodules in addition to the genes in the “main” module. The submodules are not distinct enough from the main module to be identified as separate modules by the module identification procedure.

**Figure 3:**
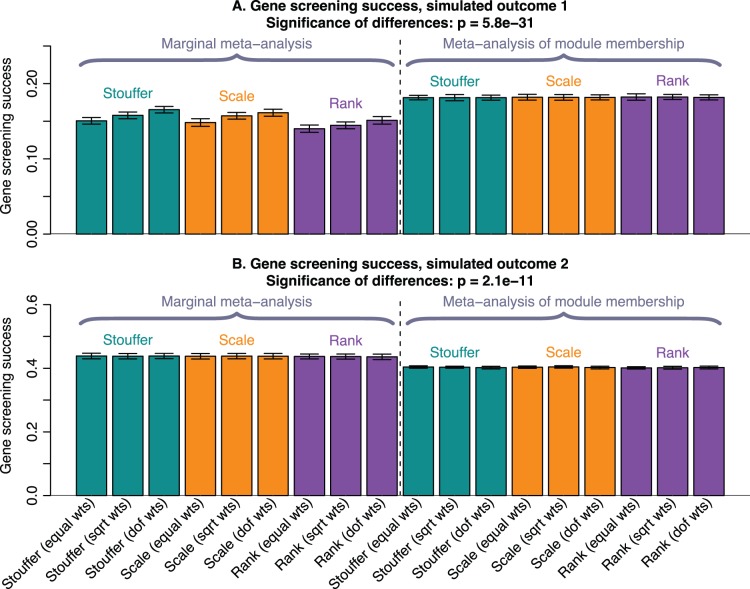
Simulation studies of gene screening success of meta-analysis methods. The barplots show validation success of the various meta-analysis methods in simulated data with 2 different traits. Continuous clinical trait 1 is weakly related to a module eigengene that may, in real data, represent the state of a pathway. In this case meta-analysis of module membership outperforms marginal meta-analysis in identifying validated genes. In contrast, clinical trait 2 is simulated to be strongly correlated with the eigengene of a small submodule of one of the identified modules. Here marginal meta-analysis outperforms meta-analysis of module membership. Analogously to Figure 2, each bar summarizes the gene screening success of the corresponding meta-analysis methods for each of the simulated traits. For each meta-analysis method we rank the genes based on the method and retain the top 50 genes. We define gene screening success as the average correlation of these top 50 genes with the trait of interest in an independent validation data set, averaged over the validation sets in each application. Each bar represents the gene screening success; error bars give the corresponding standard deviation of the observed gene–trait correlations in the top 50 genes.

We simulate two quantitative traits. The first trait is simulated to be weakly associated with a module that in real data may represents a pathway or process. Specifically, we simulate a weak association (correlation 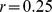) with the module eigengene. Therefore, associations of the trait with the individual module genes are noisy, but the most associated gene should also be highly correlated with the eigengene, i.e., have high module membership. In this simulation (and probably in real data involving preserved modules), the module membership is better preserved than the gene-trait associations. Hence, selecting intramodular hubs (meta-analysis of module membership) outperforms the standard marginal meta-analysis in this simulation study (Figure 3A).

The second quantitative trait is simulated in a similar fashion, but with two important differences. First, the trait is simulated to be related to one of the submodules of the large module 1. Second, the (sub-)module–trait association is simulated to be stronger. In this case the large module 1 will be selected as the module most highly associated with the clinical trait. However, because (1) the genes with the highest module membership in the large module are not the ones most strongly correlated with the trait and (2) the signal (i.e., gene–trait correlation) is strong, selection by module membership is not the optimal strategy, and marginal meta-analysis outperforms meta-analysis of module membership (Figure 3B).

## Discussion

This article describes the following results relevant to the question of when hub gene selection is preferable to selection by marginal association with a trait. First, we show that hub genes defined with respect to whole-network connectivity (Equation 14) are often uninteresting in correlation networks constructed from data from higher organisms. This finding underscores the importance of focusing on intramodular hubs. Revisiting network analyses in lower organisms (for example, yeast) reveals that even for lower organisms intramodular hubs are more essential than whole-network hubs [7].

Second, we show that selecting intramodular hubs in a relevant module often leads to gene lists with cleaner biological annotation (typically evaluated using functional enrichment analysis). This is relevant for studying candidate biological processes associated with the trait of interest.

Third, we show that marginal meta-analysis leads to superior validation success (reproducibility) of gene–trait associations in 2 out of 3 applications. This supports the claim that standard marginal approaches are in general more suitable for biomarker discovery. An exception to this rule is the adenocarcinoma application where selecting biomarkers based on module membership (hub gene status) with respect to a cell proliferation module leads to superior validation success in independent data sets. To a cancer biologist it is hardly surprising that proliferation genes correlate with cancer outcomes, which is why cancer studies such as [6], [19] emphasized their focus on intramodular hub genes as opposed to whole-network hubs.

While biologically intuitive, it is difficult to understand statistically why selecting intramodular hubs as biomarkers can outperform selection by marginal association. To address this issue, we report simulation studies that describe a scenario where marginal associations are weak and noisy, while module membership (and hub gene status) are strongly preserved between training and validation data sets. In this simulation scenario, marginal meta-analysis statistics are prone to finding false positives while module membership with respect to preserved modules carries more reproducible information.

Methods that evaluate the biological enrichment of gene lists need to be careful to avoid a bias stemming from first looking at the enrichment results before choosing an enrichment category as gold standard. For example, a severe bias in favor of a consensus module screening method would result if one first identified the most significant GO category for the consensus module and then used this GO category as gold standard for assessing the biological signal in a gene list produced via a standard marginal meta-analysis technique. Our study avoided this kind of bias by focusing on confirmed GO categories that were known a priori from the literature and selecting modules by the correlation between their module eigengene and the trait. Specifically, in our lung cancer application (Application 1) we chose the GO term “cell cycle” since it is well known that genes whose over-expression is associated with shorter survival time are often enriched with cell cycle genes [45], [46]. This reflects that a growing, proliferating tumor is often associated with shorter patient survival [47], [48]. The relevant module (module 2) was chosen because its eigengene had the highest correlation with survival time across the lung cancer data sets (Figure S2 in Text S1). Finally, one can also compare the highest enriched terms for the relevant consensus module (detailed in Table S1) to the highest enriched terms for the genes identified by marginal meta-analysis (Table S2). In this case, the top enriched terms are very similar (all relate to cell cycle) but the enrichment of the genes selected by meta-analysis of module membership is much higher. Hence, even if one were to select the gold standard by looking at the enrichment of genes selected by marginal analysis, meta-analysis of module membership would still lead to much higher enrichment.

Application 3 (total cholesterol in mice) highlights additional challenges that arise when there is no clear gold standard and multiple modules are strongly associated with a trait. Our chosen gold standard (immune system processes) was captured by the most significantly associated module. But there are likely other functional categories important for TC that may be captured by other strongly associated modules. In this sense, applications without a clear gold standard and/or with multiple trait associated modules require judgment calls when comparing network methods with standard marginal methods.

### Discussion of Marginal Meta-analysis Methods

The marginal meta-analysis methods discussed in this article include standard meta-analysis statistics such as Stouffer’s method which is based on combining Z statistics (or equivalently using the inverse normal method), as well as rank-based meta-analysis techniques that aggregate ordinal measures of variable importance. Rank-based methods can be attractive when (1) a large number of variables is available and (2) when significance tests in each of the underlying data sets are difficult (e.g. due to the presence of hidden structure in the data that can lead to over- or under-dispersion). In particular, rank-based methods are ideally suited to meta-analysis of network centrality (or other network indices) since it is often difficult to define and calculate statistical significance for such quantities. For example, while module membership in a correlation network can be measured by correlation for which a p-value is easily calculated, in a general network this is typically not the case.

Many rank-based meta-analysis methods have been described in the literature, for example [2], [57]–[59]. Most of these methods rely on computationally expensive permutation tests. In contrast, our rankPvalue approach (and R function) makes use of computationally fast asymptotic testing procedures that are either based on the convolution of uniform distributions (giving rise to the Rank method) or rely on the central limit theorem (giving rise to the Scale method, Equation 5). Disadvantages of all ranking based meta-analysis approaches include that they require multiple data sets (at the very least 4 data sets [37]) and a large number of variables (hundreds if not thousands).

Our applications as well as simulations indicate that the rankPvalue approach (both Scale and Rank method) leads to results that are broadly comparable to those of Stouffer’s method when these methods use the same choice of weights for the data sets. Our results provide no conclusive guidance as to which of the three weight choices for data sets (constant, degree of freedom, or square root weights) lead to highest validation success. Although the theoretically optimal choice, under certain assumptions, are the square root weights [26], the assumptions underlying this result may not be fulfilled in practice.

While the choice of meta-analysis weights clearly has a significant effect on the resulting gene lists, it does not affect the main conclusions of our applications and simulations: the choice of standard marginal meta-analysis versus the selection of intramodular hubs in consensus modules has a much more pronounced effect than the choice of the weighting scheme.

### Discussion of Hub Gene Selection Methods

The selection of intramodular hub genes requires some judgment. Even in case of a single data set (and a single network) the data analyst has to decide between intramodular connectivity 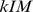 (Equation 15) and module membership 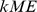 (Equation 19). Fortunately, one can show theoretically and empirically [17], [18], [60] that these two measures are often strongly related. This justifies our focus on a single measure, 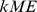. Compared to intra-modular connectivity 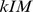, module membership 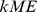 has the advantage of being defined via a correlation, which makes the calculation of the associated p-values straightforward. In turn, this makes 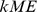 suitable for standard meta-analysis methods for correlation tests.

In case of a consensus network analysis based on multiple independent data sets, the situation becomes more complicated. Since each data set corresponds to a network, one arrives at one measure of 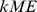 per data set. To combine these correlation measures across networks, i.e., to arrive at a consensus measures of 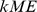, one can again apply meta-analysis techniques to the correlation tests used for defining 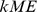. As part of this article, we evaluated the performance meta-analysis methods applied to 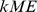 across all input data sets. With the exception of the adenocarcinoma application where Stouffer’s method outperforms rank-based meta-analysis, all methods considered here perform similarly.

Marginal meta-analysis simply selects genes with the most significant meta-p-values; these genes are not necessarily highly correlated to one another. In contrast, network screening methods that select intramodular hub genes often result in gene lists whose members have relatively high pairwise correlations.

### Limitations

Our study has several limitations. First, our applications involve correlation networks in higher organisms. In other types of networks, e.g. information networks, protein-protein interaction networks in lower organisms, and others, whole-network hubs are clearly very important [14], [15], [61], [62].

Second, our analysis only considered a limited number of standard marginal meta-analysis approaches and network based approaches. While is is likely that our results generalize to other marginal methods as well, space limitations do not permit a comprehensive evaluation of the many approaches described in the literature. In particular, we did not evaluate hybrid approaches that study network connections among known biomarkers [63].

Third, both rank based meta-analysis methods have the limitation of, in general, requiring multiple (at the very least 4) data sets. In particular, the asymptotic approximation at the heart of the Rank method breaks down when dealing with fewer than 4 independent data sets. The number of data sets required for the Scale ranking method depends on the distribution of the underlying ordinal variables: while it (and the central limit theorem) do not assume normally distributed ordinal variables, fewer data sets are needed if approximate normal applies.

Fourth, we have formulated our comparisons for the case when there is a single trait-related module, i.e., when hub genes are only selected on the basis of a single module. In some applications there may be several trait-related modules (for example, one positively and one negatively related to the trait) and the data analyst needs to decide which module to choose. In practice, data analysts would of course consider functional enrichment with respect to gene ontology categories or cell marker in order to find a biologically credible module.

Fifth, selection of intramodular hubs crucially depends on identifying a relevant trait-related consensus module across possibly very different data sets. Meta-analysis of module membership can only be successful if the module is present in all analyzed data sets (i.e., the module is robust) and if its relationship to the clinical trait is reproducible. While many published article describe trait-related modules, it is by no means guaranteed that trait related consensus modules can be found. In particular, if the input data are measured on different platforms or are incompatible for some other reason, then consensus modules may not exist. It is often useful to assess the compatibility of the input data sets by studying the concordance of mean expression, whole-network connectivity [8], [64], and to carry out module preservation analysis [65]. In our situation, module preservation analysis was not needed since relevant consensus modules are present in each application.

Sixth, our focus on intramodular hubs should not mislead the data analyst to ignoring prior knowledge about module genes or to ignore complementary data. If regulatory relationships are of interest, transcription regulators (e.g., a transcription factor) of modules may be much more worthwhile targets for follow up studies than intramodular hubs.

Our results have no direct relationships to the dissection of regulatory networks. Important articles describe and evaluate regulatory network inference procedures, e.g., [66], [67]. In particular, we do not consider how to integrate co-expression, protein-protein interaction, and other types of data. We emphasize again that prior biological knowledge and complementary data are invaluable for prioritizing genes for follow-up studies.

Seventh, our results apply to correlation networks which are undirected graphs. There is a vast literature on network inference procedures for constructing directed and causal network models.

While our results show that network based meta-analysis (referred to as consensus module analysis) can be superior to standard marginal methods for identifying relevant biological processes, it is worth emphasizing that each application and data set will require a careful evaluation of all available analysis options.

## Methods

### Standard Meta-analysis Methods

Meta-analysis is a well-established technique for aggregating data from separate studies [33]–[36], [68], [69]. It is increasingly being used to more fully utilize the rapidly accumulating high-throughput biological data sets (e.g., gene expression, methylation and genotyping), since pooling of raw data from high-throughput experiments is often not feasible. A typical use of meta-analysis in genomics is to combine several studies in which one evaluates the association of a clinical trait (for example, disease status or survival time) with, say, gene expressions measured by a high-throughput method. Multiple methods were developed specifically for marginal meta-analysis of gene expression data [58], [59], [70]–[73] and a comparison was performed, e.g., in [74]. Discussions of issues that arise in meta-analysis of gene expression data, as well as references to multiple applications, can be found, for example, in [75]–[78]. Here we give a brief overview of the meta-analysis methods used in this article; a full review of the many methods proposed in the literature is beyond the scope of this article.

One of the earliest meta-analysis techniques was proposed by Fisher [68]. Given 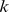 independent statistical tests and their associated p-values 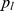, one forms the test statistic
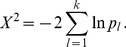
(1)


Under the null hypothesis 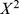 follows the 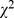 distribution with 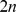 degrees of freedom. This method can be generalized [36] by defining a test statistic 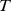 as
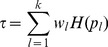
(2)where 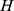 is a suitable function and 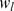 are (non-negative) weights for each study. For several different choices of 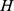 and 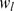 the null distribution of 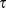 is known. Careful choice of 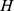 and 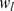 can lead to a meta-analysis test with better power. We now discuss three choices of 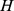 and 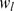 that are used in this article.

The first choice, also known as the inverse normal method, was proposed by Stouffer et al. [33]. It is based on individual test Z statistics 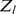 that are obtained from the corresponding p-values using the inverse normal distribution. One then forms the test statistic
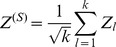
(3)that under the null follows the normal distribution 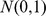. This test is referred to as Stouffer’s test (with equal weights).

Stouffer’s method was generalized to allow different weights for the individual tests by Mosteller and Bush [69] and Liptak [34]. Given positive weights 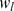, one forms the weighted Z statistic
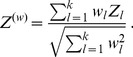
(4)


The statistic 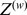 again follows the standard normal distribution 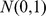. The optimal choice of weights depends on effect size and standard error of its estimate in each study [36]. Assuming that samples in all studies have been drawn randomly from the same pool the theoretically optimal weight choice is for 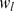 to be proportional to the square root of the number of samples in each study, 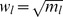 [34]–[36]. We call this method Stouffer’s methods with square root weights. In this work we also investigate setting 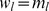 and refer to this method as Stouffer’s method with degree of freedom (dof) weights. (We approximate the degrees of freedom in each study by the number of samples.).

### R Software Implementation

Marginal meta-analysis methods described in this article are implemented in the function metaAnalysis that is part of the updated, freely available package WGCNA for the R language and environment. Although our examples involve only continuous traits, the function can also analyze binary traits using t-test or Kruskal–Wallis rank sum test. Users can specify custom weights for the individual data sets as well as the 3 standard choices of weights described here. Robust correlation (specifically, the biweight mid-correlation [79], [80]) can be used to efficiently suppress potential outlier measurements. Optionally, Scale and Rank meta-analysis can also be performed automatically making the function metaAnalysis a convenient “one-stop” option for calculating a multitude of marginal meta-analysis statistics.

### The rankPvalue Meta-analysis Method and R Function

Stouffer’s method requires as input Z statistics that, under the null, are normally distributed with mean 0 and variance 1. While Z statistics are easily computed for many standard association tests, they are not available for many common network indices such as whole-network or intra-modular connectivity. Even when Z statistics can be computed, their actual null distribution may differ from the theoretical 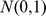 distribution because of technical effects or hidden relationships among samples such as population stratification. Therefore, we now describe a method, called rankPvalue, that uses as input a general ordinal measure of variable importance. There are 2 variants the rankPvalue method presented in turn below.

The Rank variant first ranks each variable (labeled by the index 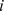) separately in each set (labeled by the index 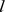) based on the input statistics. The ranks 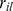 that range from 1 to the number of non-missing observations 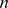 are then converted to percentile ranks 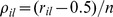. Under the null, the observed percentile ranks follow a uniform distribution over the allowed values which can be approximated by a continuous uniform distribution. The test statistic is then formed as the weighted sum
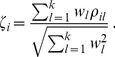
(5)


The formula (5) is analogous to Equation 4, and in this article we use the same weights as for Stouffer’s method. Under the null hypothesis that there is no relationship between the rankings of the input statistics between the individual data sets, the test statistic 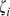 follows a distribution that is given by the convolution of uniform distributions. Using the Central Limit Theorem, one can then argue argue that the row sum test statistic follows asymptotically a normal distribution. It is well-known that the speed of convergence to the normal distribution is extremely fast in case of identically distributed uniform distributions [37]. Even when there are only 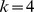 input studies, the difference between the normal approximation and the exact distribution is negligible in practice.

The Scale variant follows a logic similar to the Rank variant, but instead of converting each variable importance to a rank, it scales the variable importance measures in each input data set to mean 0 and variance 1. The meta-analysis test statistic is calculated according to Equation 4 with the same weights that are used for Stouffer’s method. The Central Limit Theorem again guarantees convergence of the null distribution of the meta-analysis statistic 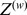 to 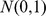, but in general the speed of convergence may not be as fast as that of the rank-based meta-analysis statistic 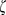 (Equation 5).

Both Rank and Scale variants are implemented in the function rankPvalue which is also included in the WGCNA package for R. The input of the function is a variable importance measure from several independent data sets and optional weights for each data set. The user can choose whether to calculate meta-analysis p-values using the Rank, Scale, or both variants. As an added convenience, the function can also calculate local False Discovery Rate estimates (q-values) [81], [82].

### Weighted Correlation Network Analysis

Here we provide a brief overview of Weighted Correlation Network Analysis [3], [63]. A general network consists of nodes and pair-wise connections among the nodes. In unweighted networks, the connections are either present or absent (equivalently, connection strengths are 1 or 0). In weighted networks, each pair of nodes is connected and the connection strength can take any value in the interval [0,1]. In our applications, the nodes represent measured variables such as gene expression or methylation profiles.

Correlation networks are constructed from numeric data that represents multiple measurements (“samples”) of a set of variables (for example, gene expression, protein levels, etc). The measurements are assumed to be organized in a matrix 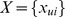 where the column index 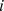 (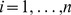) corresponds to variables, and the row index (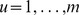) corresponds to sample measurements. We refer to the 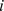-th column 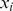 as the 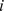-th *node profile* across 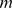 sample measurements. For example, if 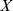 contains data from expression microarrays, the columns correspond to genes (or microarray probes), the rows correspond to microarrays, and the entries report transcript abundance measurements. Correlation networks based on gene expression data are often referred to as gene co-expression networks.

We consider undirected networks that are fully specified by their adjacency matrix, a square symmetric matrix 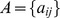, whose element 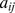 encodes the connection strength between variables 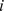 and 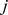. Formally, an adjacency matrix is required to be square and satisfy the following properties:
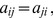
(6)

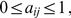
(7)

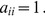
(8)


In correlation networks the adjacency is constructed from the pairwise correlations 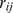 of node profiles, 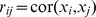.

An important choice in the construction of a correlation network concerns the treatment of strong negative correlations. In *signed networks* negatively correlated variables are considered unconnected. In contrast, in *unsigned networks* variables with high negative correlations are considered connected (with the same strength as variables with high positive correlations) [3], [84]. A signed weighted adjacency matrix can be defined as follows
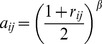
(9)and an unsigned adjacency by



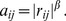
(10)The parameter 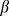 is chosen such that low correlations that typically arise due to noise are sufficiently suppressed. A general heuristic procedure for choosing 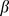 is described in [3]. Values of 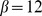 for signed networks and 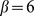 for unsigned networks often work well. The choice of signed vs. unsigned networks depends on the application; both signed [27], [84] and unsigned [6], [22], [24] weighted gene networks have been successfully used in gene expression analysis.

We find it convenient to define two functions (transformations) of adjacency matrices. First, the Topological Overlap Matrix (TOM) [85], [86] is defined as
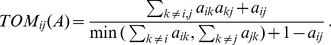
(11)


It can be shown that the matrix 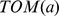 is also an adjacency matrix, i.e., 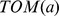 also satisfies properties (6) – (8).

Second, the dissimilarity matrix corresponding to an adjacency 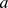 is defined as
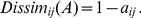
(12)


A major step in many network analyses is to identify modules. We define modules as groups of highly correlated (or, in network language, strongly inter-connected) variables. To this end, one can define a pairwise node dissimilarity measure that can be used as input in a clustering procedure. In our examples we use the dissimilarity given by
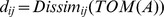


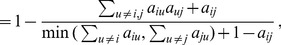
(13)as input to average-linkage hierarchical clustering [87]. Modules correspond to branches of the resulting hierarchical clustering tree (dendrogram) and are identified using the Dynamic Tree Cut procedure [88].

### Network Hubs: Nodes with High Connectivity

In many networks, from networks of airline connections to the Internet to some biological networks, the most important nodes tend to be those that have a large number of connections [14], [15], [62]. More formally, given a network specified by an adjacency matrix 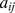, the whole-network connectivity 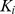 of node 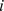 is defined as
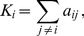
(14)that is, as the sum of the connection strengths to all other nodes in the network. Nodes with high whole-network connectivity (relative to other nodes in the network) are called whole-network hub nodes (hub genes in gene networks). Whole-network connectivity and whole-network hub nodes are often referred to simply as connectivity and hub nodes.

While whole-network connectivity is important in many contexts, our results and results of others (e.g, [89]) indicate that nodes (for example, genes) important for particular functions in large, complex networks are often not among the whole-network hubs. However, often a sub-network of the whole network is associated with the particular function, and nodes most relevant for the function are often highly connected *within the relevant sub-network*. In this work, we identify the relevant sub-networks as modules that are associated with the studied clinical trait. Correspondingly, we define the intra-modular connectivity 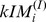 of node 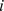 within a module labeled 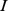 as
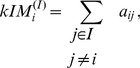
(15)that is, as the sum of the connection strengths within the module 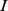. Nodes with high intra-modular connectivity are called intra-modular hub nodes.

### Eigennode Summarizes a Correlation Module

Many module construction methods lead to correlation network modules comprised of highly correlated variables. For such modules one can summarize the corresponding module vectors using a representative variable, in network terminology also known as a representative node profile. To define the representative profile of a module, we use the Singular Value Decomposition (SVD) of the **standardized** module matrix [90]. The matrix of the module 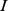 is denoted by 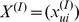, where the index 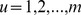 corresponds to samples and the index 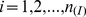 corresponds to the module variables (nodes of the network). For ease of notation, we will drop the module index 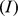; the reader should keep in mind that the discussion below is specific to a particular module. In the first step of defining the module eigennode, we standardize each variable (column) in 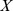 to mean 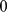 and variance 1. This important step ensures that the definition of the eigennode is independent of the overall scale of each column that can be affected by various technical factors, for example the overall scale of microarray expression profiles is affected by microarray probe sensitivity to individual transcripts. The singular value decomposition of the standardized module matrix 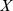 is denoted by
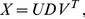
(16)where the columns of the orthogonal matrices 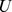 and 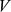 are the left- and right-singular vectors, respectively. Specifically, 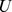 is an 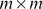 matrix with orthonormal columns, 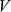 is an 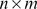 orthogonal matrix, and 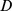 is an 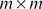 diagonal matrix of the singular values 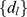, 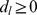. The matrices 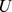 and 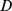 are given by



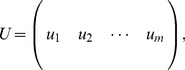
(17)

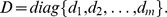



We assume that the singular values 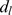 are arranged in non-increasing order. Adapting terminology from [8], [24], [25], [90], we refer to the first column of 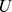 as the *module eigennode* (also known as module eigengene in gene co-expression or co-methylation networks):
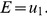
(18)


Since the orientation (i.e., sign) of each singular vector is undefined, we fix the orientation of each eigennode by constraining it to have a positive correlation with the average gene expression across module genes. Our definition of the eigennode assumes that the highest singular value 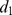 is non-degenerate module matrix 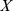 is non-degenerate, that is, we assume that the singular values 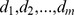 are In practice, we find that the module eigennode typically explains more than 50% of the variance of the module expressions.

We note that one can also define the eigennode using principal component analysis (PCA). In PCA, one performs an eigenvalue and eigenvector analysis of the sample covariance matrix 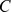 whose element 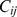 is the covariance of the node profiles 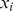 and 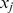, that is 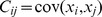. The resulting eigenvalues 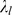 and eigenvectors 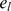 satisfy 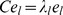. Because the covariance matrix is symmetric non-negative definite, all eigenvalues 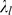 are real and non-negative, 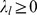, and can be ordered in a non-increasing order (i.e., 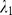 is the largest eigenvalue). The first principal component 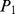 is then defined as 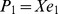. Because the module matrix 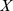 is scaled to mean 0 and variance 1, one can show that 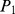 and the first left-singular vector 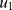 (Equation 17) differ only by a constant, 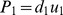. Since the overall scale of the module summary profile in correlation networks is irrelevant, the first principal component 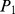 provides an equivalent summary as the eigennode 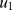.

We now briefly comment on the right-singular vectors 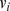. Recall that the first left-singular vector 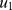 can be interpreted as the summary of the profiles of all variables (e.g., expression profiles), in the module. In contrast, the first right-singular vector 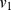 can be interpreted as a summary of the expression profiles of the samples. The right-singular vectors can be utilized to perform signal-balancing; the details are beyond the scope of this article and we refer the interested reader to Section 6.1.1 in the book [18] and references therein.

### Eigennode-based Fuzzy Module Membership Measure

The module eigennode 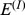 can be used to define a quantitative measure, denoted 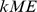, of module membership of variable 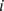 in module 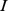 [17]:
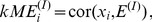
(19)where 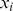 is the profile of node 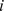. The module membership 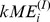 lies in 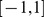 and specifies how close node 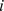 is to module 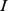. The quantity 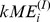 is sometimes referred to as signed module eigengene-based connectivity [24], [25]. In gene co-expression networks, module membership and intra-modular connectivity tend to be very highly correlated due to approximate factorizability of the module sub-networks [17], [60].

### Eigennode-based Measure of Module–trait Association

The module eigennode also gives rise to a convenient measure of module–trait association. Given a quantitative trait 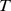 and a module labeled 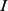 with eigennode 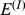, we define the module eigennode significance 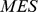 (sometimes also called module significance) as the correlation of the trait and the eigengene,
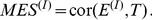
(20)


Module eigennode significance lies in 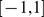. Values close to 1 (−1) indicate a module very strongly positively (negatively) associated with the trait, while values close to 0 mean the linear association is weak. Because the module significance is defined as a correlation, it is straightforward to quantify its statistical significance by the corresponding correlation test p-value. Hence, module eigennode significance is well-suited for meta-analysis using Stouffer’s method as well as our Scale and Rank modifications.

### Consensus Modules

Advantages of meta-analysis and related techniques have long been recognized in network analysis. Several sophisticated algorithms for finding commonly-occurring subnetworks (sometimes referred to as modules) have been developed, for example [2], [91]–[95]. *Consensus modules* are defined as sets of highly connected nodes that can be found in multiple networks. A comparison and evaluation of different approaches for finding consensus modules is beyond our scope and we refer the reader to the literature [38], [93]–[95].

Since our focus is the utility of using consensus modules for selecting genes, we restrict our attention to a single consensus module detection approach [38] within the WGCNA framework. Consensus modules are identified using a suitable *consensus dissimilarity* that is used as input to a clustering procedure, analogously to the procedure for identifying modules in individual sets. To simplify our discussion we introduce the following component-wise quantile function for a set of 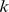 matrices 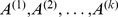:
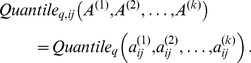
(21)


Thus, each component of the quantile matrix is the given quantile (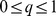) of the corresponding components in the individual input matrices. Using this notation, we define the consensus network corresponding to input networks 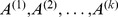 and a quantile 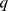 as
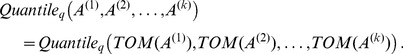
(22)


When 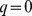, i.e., the quantile is the minimum, the consensus network has a very simple interpretation: two variables are connected with the strength that is common to all input networks (hence the name “consensus”).

To identify consensus modules, we use the standard module identification procedure with the dissimilarity
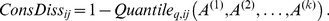
(23)


We emphasize again that this procedure is meaningful only when the variables of the input networks are the same.

### Meta-analysis of Module Membership in Consensus Modules

Once consensus modules are identified, their eigengenes (Equation 18) can be calculated in each input data set 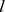. Specifically, denote the eigengene of module 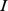 in set 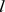 by 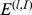. For each node 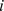 we then have 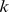 measures of module membership, namely
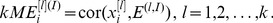
(24)


Several alternative ways of summarizing the 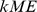 measures are possible. First, since 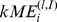 is defined as a correlation, one can turn it into a Z statistic and use the standard meta-analysis techniques described above (Equations 3 and 4), as well as our Scale and Rank modifications. We use these methods in the reported results.

For completeness, we also describe two alternatives to meta-analysis of Z statistics derived from individual 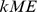 values that are simpler but in general do not perform as well. First, one can apply the consensus approach and define consensus module membership
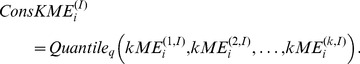
(25)


Second, one can also define the (weighted) mean 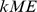. Given weights 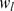 for each data set, 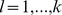


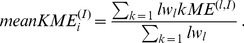
(26)


The weights can be the same as those were used to define various versions of the meta-analysis Z statistics, although this is not a requirement.

Meta-analysis of consensus module membership is implemented in the function consensusKME that is also included in the WGCNA package. This function provides an interface similar to that of the function metaAnalysis, including various choices of individual set weights, optional automatic calculation of Scale and Rank meta-analysis, and optional use of a robust correlation measure.

### Adenocarcinoma Data Sets and Network Analysis

We have downloaded 8 independent cancer data sets: 4 data sets [40] measured on Affymetrix U133A microarrays that comprise 162, 69, 73, and 89 samples, respectively; 51 samples [41] measured on Affymetrix U133plus2 microarrays; 91 samples [42] measured on Agilent Whole Human Genome oligo DNA microarray G4112F; 81 samples [43] measured on Agilent Homo Sapiens 21.6K custom array; and 49 samples [44] measured on Agilent-012391 Whole Human Genome Oligo Microarray G4112A. The numbers of samples in each data set reflect restriction to Adenocarcinoma (AD) where applicable and our removal of possible outlier samples.

Because the microarray probes differ between the 5 platforms present in this study, we used the aggregating approach described in [96] (implemented in the collapseRows function) to “collapse” the probe-level expression data to gene-level expression data. We then retained only expression profiles of the 8655 genes that are represented on each of the 5 platforms.

The consensus TOM was defined as the consensus (Equation 22) of the individual TO matrices with percentile 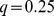 (i.e., the quartile). Consensus modules were constructed using the approach detailed in [38] and reviewed above. This procedure resulted in 5 modules.

To measure the biological significance of each gene or module eigengene, we first calculated the survival time deviance. Then, significance of a gene or module eigengene is simply given as the correlation of the corresponding expression profile with survival deviance.

### Genome-wide Methylation Data used in Study of Aging

We analyze 3 whole blood (WB) methylation data sets and 4 region-specific brain methylation data sets. The methylation data include 190 samples from a study of Type I diabetes [49], 261 samples from healthy controls of a large cancer study [50], and 87 samples from a previous study of aging [51]. The 4 brain data sets were first reported in a study of genetics of expression and methylation in normal human brain [52]. Here we use the methylation data sets that survey genome-wide methylation across frontal cortex, temporal cortex, pons regions, and cerebellum of 150 individuals. After outlier removal, we retained 132 (frontal cortex), 126 (temporal cortex), 123 (pons regions), and 111 (cerebellum) samples. All 7 methylation data sets were assayed on Illumina Infinium HumanMethylation27 BeadChips.

We again used the 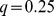 percentile to define the consensus TOM (Equation 22). The consensus module identification resulted in 41 modules. Compared to the adenocarcinoma application, the relatively large number of modules identified here is likely due to higher similarity of the individual co-methylation networks. Gene significance for each methylation probe was defined as the correlation of the corresponding methylation profile with age.

### Mouse Liver Expression Data Sets

We work with 9 independent liver expression data sets. Eight of the data sets come from 3 separate F2 mouse crosses: 2 data sets of 141 (female) and 100 (male) samples from a CAST×C57BL/6J cross denoted C×B [27]; 2 data sets of 134 (female) and 124 (male) samples from a C3H/HeJ×C57BL/6J cross on an ApoE null background denoted BxH ApoE [24], and 4 data sets of 66 (B×H female), 69 (B×H male), 63 (H×B female), and 66 (H×B male) samples from a C3H/HeJ×C57BL/6J cross on wild-type background denoted BxH wt [55]. The 9th data set of 196 male samples, called the Mouse Diversity Panel (MDP), is a genetically more diverse collection containing mice from various laboratory strains and crosses [56]. Because the 9 data sets were measured on various microarray platforms including custom Agilent two-color arrays (all F2 crosses) as well as Affymetrix HT Mouse Genome 430A Array (MDP), we again used the function collapseRows to create gene-level expression data that can be compared between the platforms.

As in our other applications, we used the 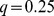 percentile to define the consensus TOM (Equation 22). The consensus module identification resulted in 11 modules. Gene significance for each gene was defined as the correlation of the gene expression profile with total cholesterol measurement in plasma.

### Simulation of Gene Expression Data

We use the data simulation functions in the WGCNA R package [83] to simulate expression data in which genes are organized into modules that group together correlated genes. We first describe the simulation of gene expression data in a single data set. To simulate an expression data set, one first chooses the number of modules and numbers of genes in each module, and a matrix that describes how the seed eigengenes of different modules should be related. Next, seed module eigengenes are generated using random, normally distributed “samples” such that their correlations approximate the given association matrix (this step is implemented in the function simulateEigengeneNetwork ). The seed eigengenes are simulated to exhibit weak to moderate correlations with one another since in empirical data we often observe that eigengenes of different clusters are correlated. For each module 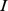, the module genes, labeled by index 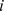, 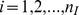, are then simulated as
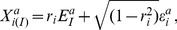
(27)where the “noise” components 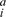 are chosen randomly and independently from 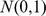, and the coefficients 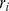 are uniformly spaced between 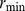 and 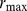. To simulate modules with strongly correlated genes, we use 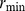 between 0.5 and 0.6, and 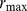 between 0.8 and 0.95. Lower values can be used to simulate modules with weaker co-expression. Most genes outside of clusters are simulated with independent expression values drawn from 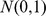, whereas a small number are simulated as “near-cluster genes” according to Equation 27, but with 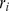 ranging from 0 to 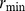. This simulation procedure is implemented in function simulateDatExpr and leads to a module structure that is generally similar to module structure observed in real data.

Since our module membership meta-analysis methods focus on consensus modules, we simulate the same module structure in all data sets, that is, all simulated modules are also consensus modules. This is conveniently achieved using the function simulateMultiExpr.

### Statistical Analysis and Code

All statistical analysis was performed using the R language and statistical environment [97], version 2.15.0. We used the network and consensus module analysis functions implemented in the WGCNA R package [80], [83], version 1.20. GO enrichment analysis within the WGCNA package is implemented in the function GOenrichmentAnalysis and relies on annotation packages provided by the Bioconductor project [98], version 2.10. (Version numbers of individual packages may differ; for example, GO annotation package GO.db as well as organism-specific annotation packages org.Xx.eg.db are version 2.7.1.) Although the qualitative conclusions reached in our analyses are robust, minor details such as exact enrichment p-values or numbers of genes in a module may differ when using different versions of Bioconductor annotation packages (due to evolving annotation databases) and the WGCNA package (due to improvements in network construction and module identification). Our pre-processing includes batch removal using the ComBat function and approach detailed in [99]. All data and analysis code are available at our web site http://genetics.ucla.edu/labs/horvath/CoexpressionNetwork/MetaAnalysis/http://genetics.ucla.edu/labs/horvath/CoexpressionNetwork/MetaAnalysis/.

## Supporting Information

Table S1 **GO enrichment of modules identified in the consensus module analysis of 8 adenocarcinoma data sets.** The table shows the 20 highest enriched terms for each module (including the improper module labeled 0). Individual columns contain module label (“Module”), module size (“Size”), rank of the term (“Rank”), Bonferroni-corrected enrichment p-value (“pEnrichment.Bonferroni”), fraction of the module genes present in the term (“Fraction”), GO ontology (“Ontology”), and GO term name (“TermName”).
(TXT)

Table S2 **GO enrichment of genes selected by marginal meta-analysis of association with survival time in 8 adenocarcinoma data sets.** The table shows the 20 highest enriched terms for the 50, 100, 200, 300, 500, and 1000 top genes selected by marginal meta-analysis. Individual columns contain number of top selected genes (“No.Genes”), rank of the term (“Rank”), Bonferroni-corrected enrichment p-value (“pEnrichment.Bonferroni”), fraction of the module genes present in the term (“Fraction”), GO ontology (“Ontology”), and GO term name (“TermName”).
(TXT)

Text S1 **This documents collects all Supporting Figures and their captions.**
(PDF)
