# Supplementary material for: When Is Hub Gene Selection Better than Standard Meta-Analysis?
Source: PLoS One. 2013 Apr 17;8(4):e61505. doi: 10.1371/journal.pone.0061505 (PMC3629234; doi:10.1371/journal.pone.0061505)
Supplement: Text S1 — This documents collects all Supporting Figures and their captions. (PDF) [file pone.0061505.s003.pdf]

# When is hub gene selection better than standard meta-analysis?
# Supporting Text S1: supplementary figures

Peter Langfelder* and Steve Horvath
*Corresponding author: peter (.) langfelder (a) gmail (.) com

This document collects the supplementary figures (and their captions) for the main article *When is hub gene selection better than standard meta-analysis?*

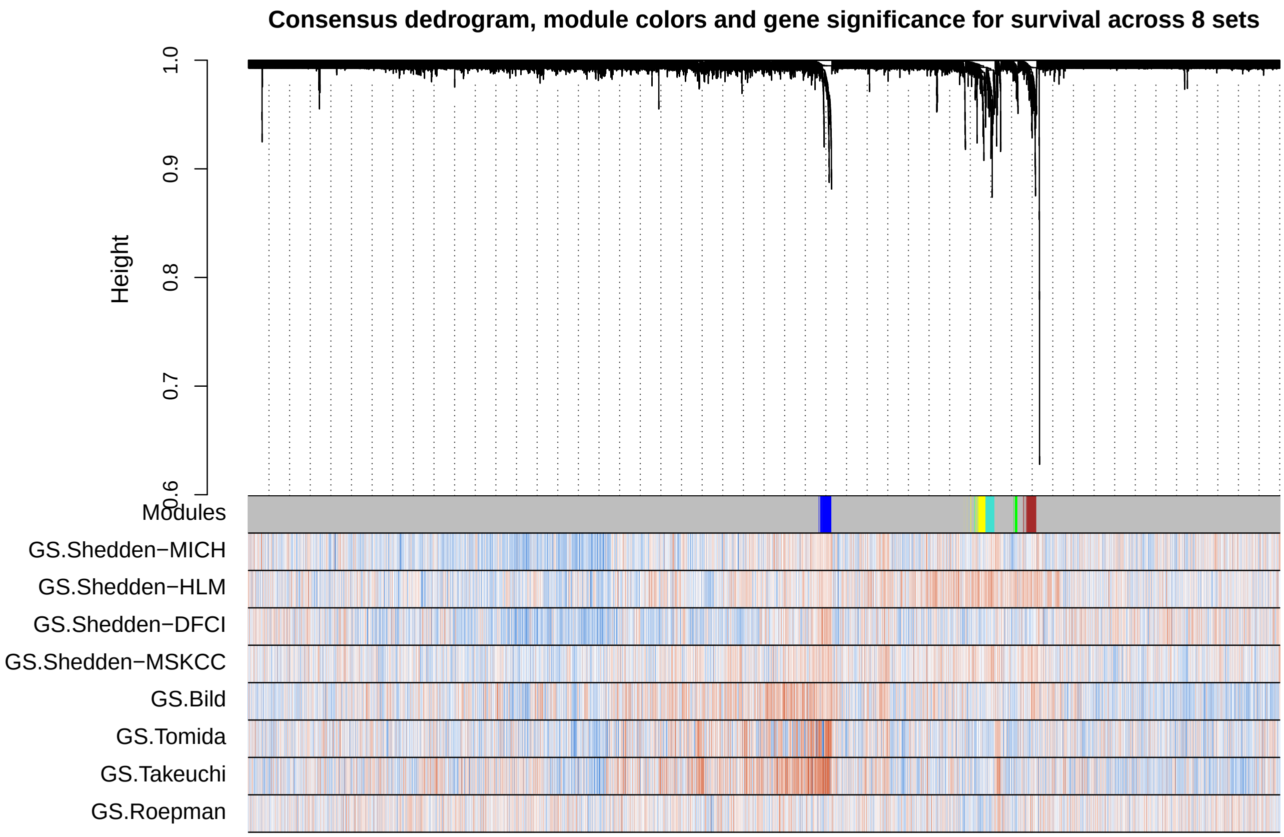


**Figure S1.** Gene clustering tree based on the consensus Topological Overlap similarity across **8 adenocarcinoma (lung cancer) data sets**. Each short vertical line ("leaf" of the tree) corresponds to a single gene. Branches of the clustering tree group together genes with high consensus similarity and hence define modules. The modules are indicated by colors below the clustering tree (line Modules). Grey color corresponds to genes not assigned to any of the modules. In this analysis, likely because of the poor reproducibility among the 8 data sets, we only find 5 small modules and most genes remain unassigned. The 8 color rows below the module indicator indicate the gene significance, defined as the correlation of survival time deviance with gene expression. Green color denotes negative correlation with survival deviance (i.e., the gene over-expression is associated with lower risk of death) while red color denotes positive correlation with survival deviance (i.e., the gene over-expression is associated with higher risk of death). This representation makes apparent the poor reproducibility of gene significance for survival time between the 8 data sets.

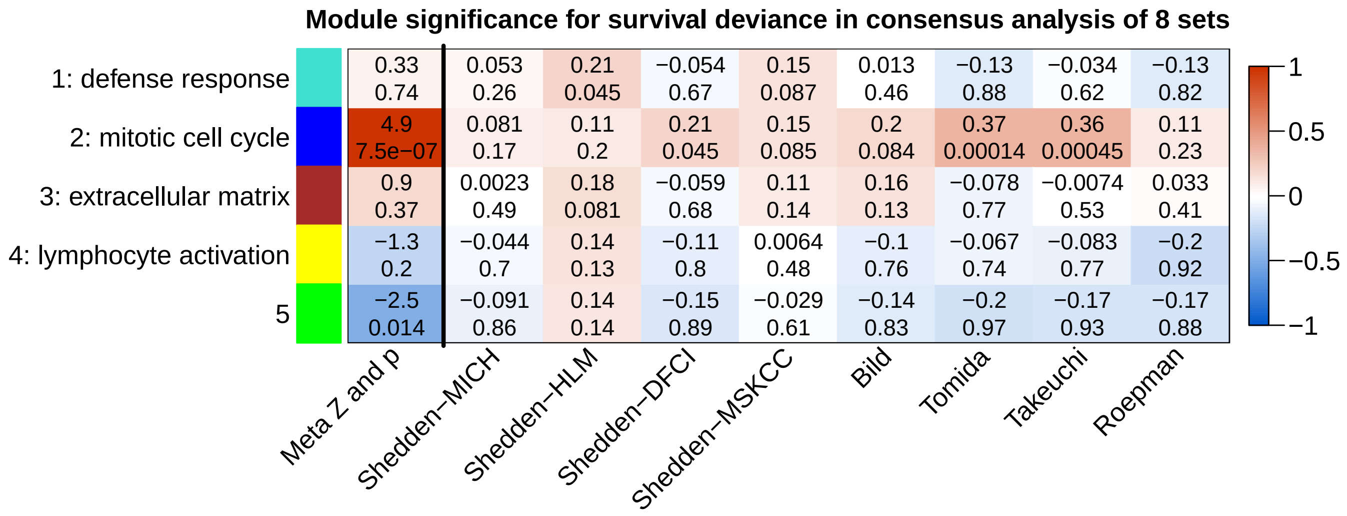


**Figure S2. Module significance for survival deviance in consensus analysis of 8 adenocarcinoma expression data sets.** Each row of this table corresponds to one module identified in the consensus module analysis. Modules are labeled by the module number and (where appropriate) a GO-derived functional label. Columns 2–9 give correlations between the survival time deviance and the module eigengenes and the associated p-values. The first column shows the meta-analysis Z statistic and the corresponding p-value. We observe that module 2 exhibits a relatively weak but consistent association with survival time deviance across the 8 data sets. The meta-analysis Z score and p-value for module 2 are significant. The significant association and cell cycle-related functional annotation We note that while module 5 also attains nominal significance at the 0.05 level, a Bonferroni correction for the number of tested modules (5) would render the p-value non-significant. Functional enrichment analysis (Supplementary Table ??) did not reveal strong enrichment in biologically plausible categories, and we do not consider this module in the following.

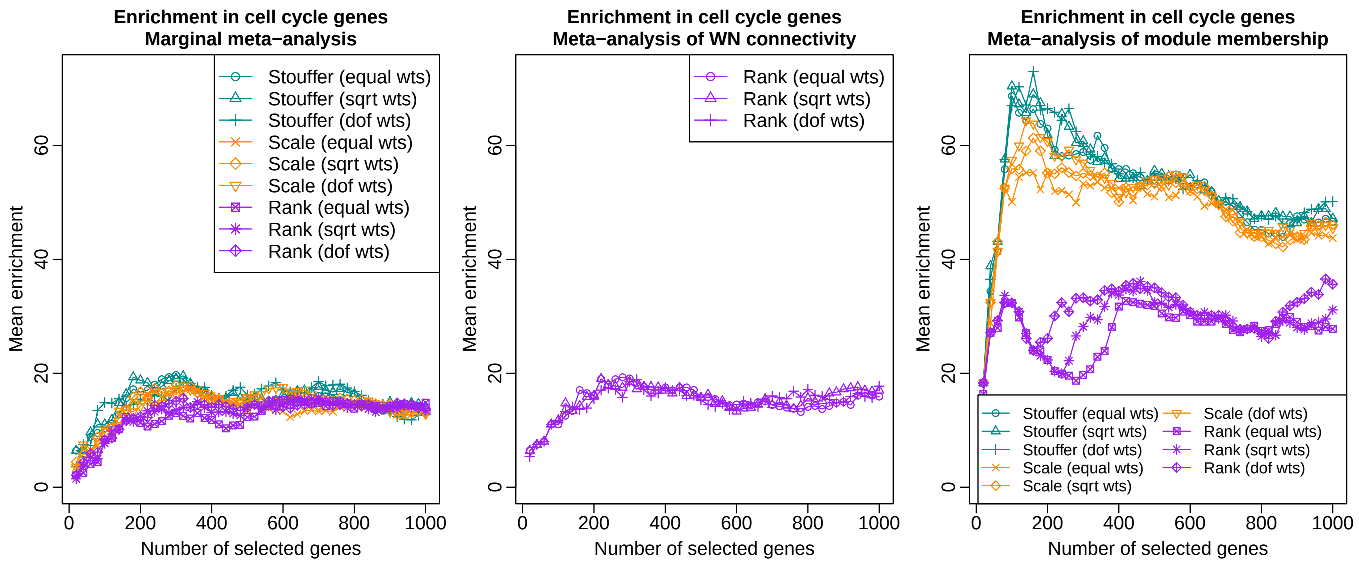


**Figure S3.** Enrichment score (defined as $-log_{10}(p)$ with $p$ being the enrichment p-value) in GO term "cell cycle" as a function of the number of top selected genes by a variety of meta-analysis (MA) methods applied to **8 adenocarcinoma (lung cancer) data sets**. The left panel shows enrichment of genes selected by 6 marginal MA methods, while the right panel shows the analogous enrichment of genes selected by MA of module membership in consensus modules. Clearly, MA of module membership in consensus modules selects genes with much higher enrichment in GO term "cell cycle".

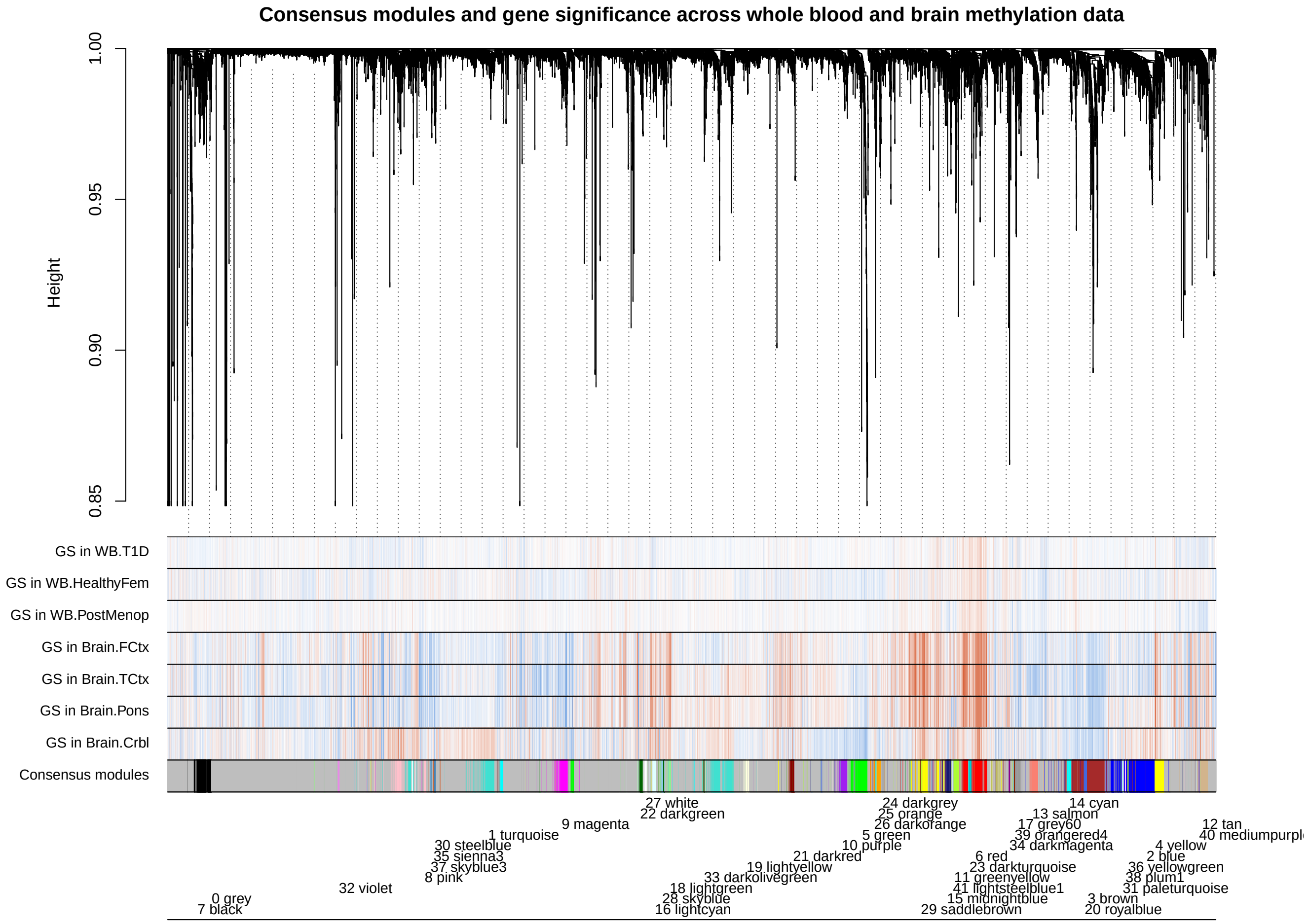


**Figure S4.** Gene clustering tree based on the consensus Topological Overlap similarity across **7 methylation data sets**. Each short vertical line ("leaf" of the tree) corresponds to a single gene. Branches of the clustering tree group together genes with high consensus similarity and hence define modules. The modules are indicated by colors below the clustering tree (line Modules). Grey color corresponds to genes not assigned to any of the modules. In this analysis we find a relatively large number (41) of modules. The 7 color rows above the module indicator indicate the gene significance, defined as the correlation of age with probe methylation. Blue color denotes negative correlation with age while red color denotes positive correlation with age.

**Consensus module eigengene significance for age**

| Module | Meta Z and p | WB.T1D | WB.HealthyFem | WB.PostMenop | Brain.FrontCtx | Brain.TempCtx | Brain.Pons | Brain.Cerebel |
|---|---|---|---|---|---|---|---|---|
| 1: intracellular | −0.82 (0.4) | −0.06 (0.4) | 0.02 (0.8) | 0.02 (0.8) | −0.25 (0.003) | −0.04 (0.7) | −0.01 (0.9) | 0.17 (0.07) |
| 2: intracellular | −1.75 (0.08) | −0.05 (0.5) | −0.07 (0.5) | −0.07 (0.3) | −0.16 (0.06) | −0.24 (0.006) | 0.09 (0.3) | 0.16 (0.1) |
| 3: intracellular | −4.84 (1e−06) | −0.03 (0.7) | −0.07 (0.5) | 0.02 (0.8) | −0.2 (0.02) | −0.36 (3e−05) | −0.36 (4e−05) | −0.26 (0.007) |
| 4: developmental process | 7.81 (6e−15) | −0.02 (0.8) | −0.08 (0.5) | 0 (1) | 0.5 (9e−10) | 0.53 (1e−10) | 0.56 (1e−11) | 0.31 (8e−04) |
| 5 | −3.24 (0.001) | 0.05 (0.5) | −0.1 (0.4) | −0.05 (0.5) | −0.11 (0.2) | −0.22 (0.01) | −0.14 (0.1) | −0.29 (0.002) |
| 6: sequence−specific DNA binding | 19.62 (1e−85) | 0.2 (0.007) | 0.25 (0.02) | 0.2 (9e−04) | 0.81 (3e−32) | 0.87 (2e−39) | 0.78 (4e−26) | 0.6 (4e−12) |
| 7 | −1.12 (0.3) | −0.08 (0.3) | −0.05 (0.6) | 0.09 (0.2) | −0.15 (0.09) | −0.16 (0.07) | −0.11 (0.2) | 0.12 (0.2) |
| 8: extracellular region | −2.51 (0.01) | −0.13 (0.08) | −0.02 (0.8) | −0.02 (0.7) | −0.07 (0.4) | −0.17 (0.06) | −0.31 (4e−04) | 0.18 (0.05) |
| 9 | −3.57 (4e−04) | 0.05 (0.5) | −0.09 (0.4) | −0.03 (0.6) | −0.15 (0.09) | −0.26 (0.003) | −0.51 (2e−09) | 0.12 (0.2) |
| 10 | −1.34 (0.2) | −0.04 (0.6) | −0.07 (0.5) | −0.01 (0.9) | −0.04 (0.6) | −0.01 (0.9) | 0.13 (0.2) | −0.31 (9e−04) |
| 11: extracellular matrix | 3.89 (1e−04) | 0.05 (0.5) | 0.11 (0.3) | −0.02 (0.8) | 0.21 (0.02) | 0.22 (0.02) | 0.09 (0.3) | 0.38 (3e−05) |
| 12: receptor activity | 1.35 (0.2) | −0.13 (0.08) | 0.05 (0.7) | −0.2 (0.001) | 0.12 (0.2) | 0.19 (0.04) | 0.35 (8e−05) | 0.28 (0.003) |
| 13 | −4.73 (2e−06) | −0.05 (0.5) | −0.1 (0.4) | −0.06 (0.3) | −0.19 (0.03) | −0.39 (8e−06) | −0.21 (0.02) | −0.14 (0.1) |
| 14 | −3.72 (2e−04) | −0.08 (0.3) | 0.08 (0.5) | −0.02 (0.8) | −0.21 (0.01) | −0.3 (7e−04) | −0.28 (0.002) | −0.04 (0.7) |
| 15 | 1.36 (0.2) | 0.06 (0.4) | 0.11 (0.3) | −0.06 (0.4) | 0.07 (0.4) | 0.21 (0.02) | 0.25 (0.005) | −0.27 (0.005) |
| 16: extracellular region | 4.34 (1e−05) | −0.1 (0.2) | −0.08 (0.5) | 0.02 (0.7) | 0.2 (0.02) | 0.26 (0.003) | 0.39 (8e−06) | 0.4 (1e−05) |
| 17 | −5.7 (1e−08) | −0.05 (0.5) | 0.11 (0.3) | −0.16 (0.01) | −0.21 (0.01) | −0.3 (8e−04) | −0.43 (9e−07) | −0.17 (0.07) |
| 18 | 13.52 (1e−41) | −0.04 (0.6) | 0.05 (0.7) | 0.02 (0.7) | 0.78 (4e−28) | 0.83 (1e−33) | 0.65 (4e−16) | 0.43 (3e−06) |
| 19 | −0.85 (0.4) | −0.02 (0.8) | −0.08 (0.5) | −0.07 (0.3) | −0.08 (0.4) | 0.2 (0.03) | −0.01 (0.9) | −0.11 (0.2) |
| 20 | −2.61 (0.009) | −0.03 (0.6) | −0.17 (0.1) | −0.04 (0.5) | −0.05 (0.6) | −0.02 (0.8) | −0.13 (0.1) | −0.24 (0.01) |
| 21: extracellular region | 1.37 (0.2) | 0.04 (0.6) | −0.12 (0.3) | −0.03 (0.7) | 0.1 (0.2) | 0.19 (0.03) | 0.35 (7e−05) | −0.26 (0.007) |
| 22: immune response | 2.08 (0.04) | −0.09 (0.2) | 0.02 (0.8) | 0.01 (0.8) | 0.2 (0.02) | 0.24 (0.006) | 0.23 (0.01) | −0.07 (0.5) |
| 23 | 8.81 (1e−18) | 0.24 (0.001) | 0.2 (0.07) | 0.19 (0.002) | 0.37 (2e−05) | 0.43 (5e−07) | 0.45 (1e−07) | 0.03 (0.7) |
| 24 | −0.34 (0.7) | −0.02 (0.8) | −0.17 (0.1) | −0.1 (0.09) | 0.13 (0.1) | 0.15 (0.09) | 0.17 (0.06) | −0.21 (0.03) |
| 25 | −2.86 (0.004) | −0.03 (0.7) | −0.14 (0.2) | −0.05 (0.4) | −0.01 (0.9) | −0.19 (0.03) | −0.07 (0.4) | −0.25 (0.009) |
| 26 | 2.13 (0.03) | 0.01 (0.9) | −0.1 (0.3) | −0.05 (0.5) | 0.22 (0.01) | 0.16 (0.07) | 0.26 (0.004) | 0.05 (0.6) |
| 27 | 0.34 (0.7) | −0.06 (0.4) | 0.02 (0.9) | −0.01 (0.8) | −0.07 (0.4) | 0.04 (0.6) | 0.13 (0.2) | 0.11 (0.3) |
| 28 | 0.16 (0.9) | −0.09 (0.2) | 0.01 (0.9) | 0.05 (0.4) | 0.08 (0.4) | −0.04 (0.7) | −0.3 (9e−04) | 0.34 (2e−04) |
| 29 | 3.16 (0.002) | −0.04 (0.6) | 0.01 (0.9) | −0.04 (0.5) | 0.26 (0.003) | 0.33 (1e−04) | 0.1 (0.3) | 0.25 (0.009) |
| 30 | −6.23 (5e−10) | 0.03 (0.7) | −0.05 (0.6) | −0.05 (0.5) | −0.34 (6e−05) | −0.46 (6e−08) | −0.35 (8e−05) | −0.31 (0.001) |
| 31 | −3.91 (9e−05) | −0.06 (0.4) | −0.08 (0.5) | −0.09 (0.2) | −0.1 (0.3) | −0.21 (0.02) | −0.38 (2e−05) | 0.02 (0.8) |
| 32 | −3.28 (0.001) | 0.02 (0.7) | 0.14 (0.2) | 0.04 (0.5) | −0.25 (0.004) | −0.25 (0.005) | −0.24 (0.007) | −0.33 (4e−04) |
| 33 | 1.15 (0.3) | −0.07 (0.3) | 0.05 (0.7) | 0.03 (0.6) | −0.13 (0.1) | 0.17 (0.06) | 0.16 (0.08) | 0.14 (0.2) |
| 34 | 2.71 (0.007) | −0.1 (0.2) | 0.2 (0.06) | −0.03 (0.6) | 0.12 (0.2) | 0.16 (0.08) | 0.47 (4e−08) | −0.02 (0.9) |
| 35 | −0.33 (0.7) | 0.01 (0.9) | 0.12 (0.3) | 0 (1) | −0.16 (0.06) | −0.12 (0.2) | −0.08 (0.4) | 0.23 (0.02) |
| 36 | −2.1 (0.04) | −0.07 (0.3) | −0.04 (0.7) | 0 (1) | −0.09 (0.3) | −0.33 (2e−04) | −0.07 (0.5) | 0.12 (0.2) |
| 37 | −5.12 (3e−07) | −0.01 (0.8) | 0.11 (0.3) | 0.02 (0.7) | −0.42 (7e−07) | −0.48 (2e−08) | −0.4 (5e−06) | −0.02 (0.8) |
| 38 | 3.8 (1e−04) | 0.12 (0.1) | 0.11 (0.3) | 0.15 (0.02) | −0.04 (0.6) | −0.09 (0.3) | 0.2 (0.02) | 0.37 (6e−05) |
| 39 | −4.48 (8e−06) | 0.1 (0.2) | 0.07 (0.5) | 0 (1) | −0.38 (9e−06) | −0.4 (3e−06) | −0.19 (0.03) | −0.34 (2e−04) |
| 40 | −7.11 (1e−12) | −0.17 (0.02) | 0.04 (0.7) | −0.19 (0.002) | −0.36 (2e−05) | −0.28 (0.002) | −0.23 (0.01) | −0.31 (8e−04) |
| 41 | 3.33 (9e−04) | 0.13 (0.07) | 0.18 (0.1) | 0.05 (0.4) | 0.11 (0.2) | 0 (1) | 0.01 (0.9) | 0.33 (3e−04) |

Color scale: 0.5, 0, −0.5

**Figure S5.** Consensus eigengene significance for age across 7 genome-wide methylation data sets. Each row of this table corresponds to one module identified in the consensus module analysis. Modules are labeled by the module number and (where appropriate) a GO-derived functional label. Columns 2–8 give robust correlations between age and the module eigengenes and the associated p-values. The first column shows the meta-analysis Z statistic and the corresponding p-value. We observe that several modules attain strong statistical significance in the meta-analysis of the 7 data sets. The most strongly associated module is module 6. The association between age and the module eigengene is consistent in all 7 data sets, lending further credence to the meta-analysis result. Hence, we focus our analysis on this module.

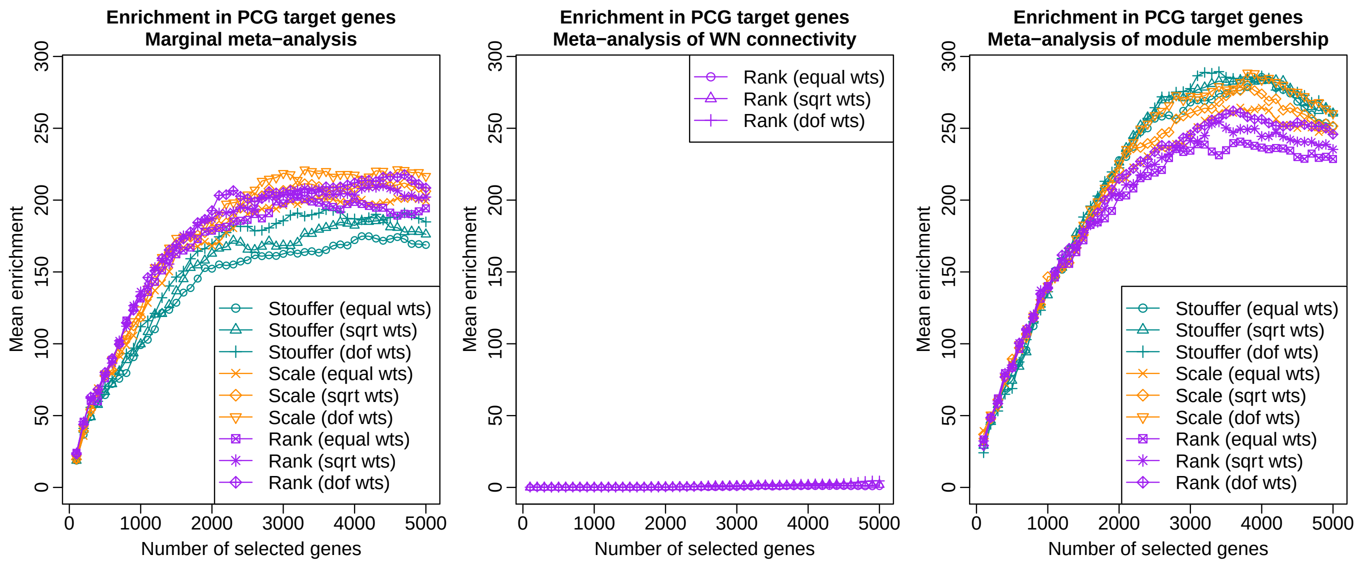


**Figure S6.** Enrichment score (defined as $-log_{10}(p)$ with $p$ being the Bonferroni-corrected enrichment p-value) in Polycomb group (PCG) target genes as a function of the number of top selected genes by a variety of meta-analysis (MA) methods applied to **7 methylation data sets**. The left panel shows enrichment of genes selected by 6 marginal MA methods, while the right panel shows the analogous enrichment of genes selected by MA of module membership in consensus modules. Although marginal MA methods results in gene sets with very strong enrichment, meta-analysis of module membership in modules selects genes with somewhat higher enrichment.

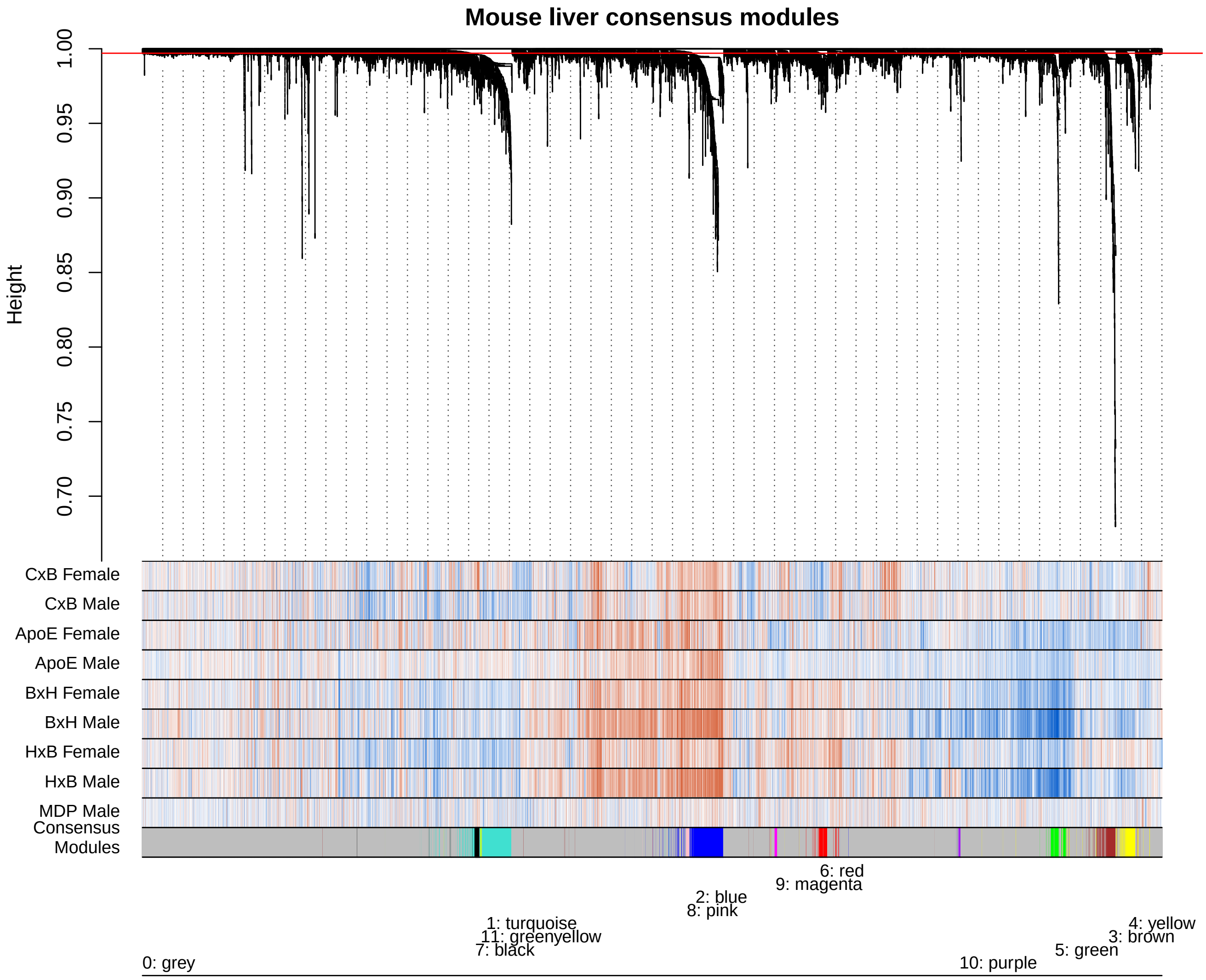


**Figure S7.** Gene clustering tree based on the consensus Topological Overlap similarity across **9 mouse liver expression data sets**. Each short vertical line ("leaf" of the tree) corresponds to a single gene. Branches of the clustering tree group together genes with high consensus similarity and hence define modules. The modules are indicated by colors below the clustering tree (line Modules). Grey color corresponds to genes not assigned to any of the modules. In this analysis we find a moderate number (11) of modules. The 9 color rows above the module indicator indicate the gene significance, defined as the correlation of total cholesterol with gene expression. Blue color denotes negative correlation with total cholesterol while red color denotes positive correlation with total cholesterol.

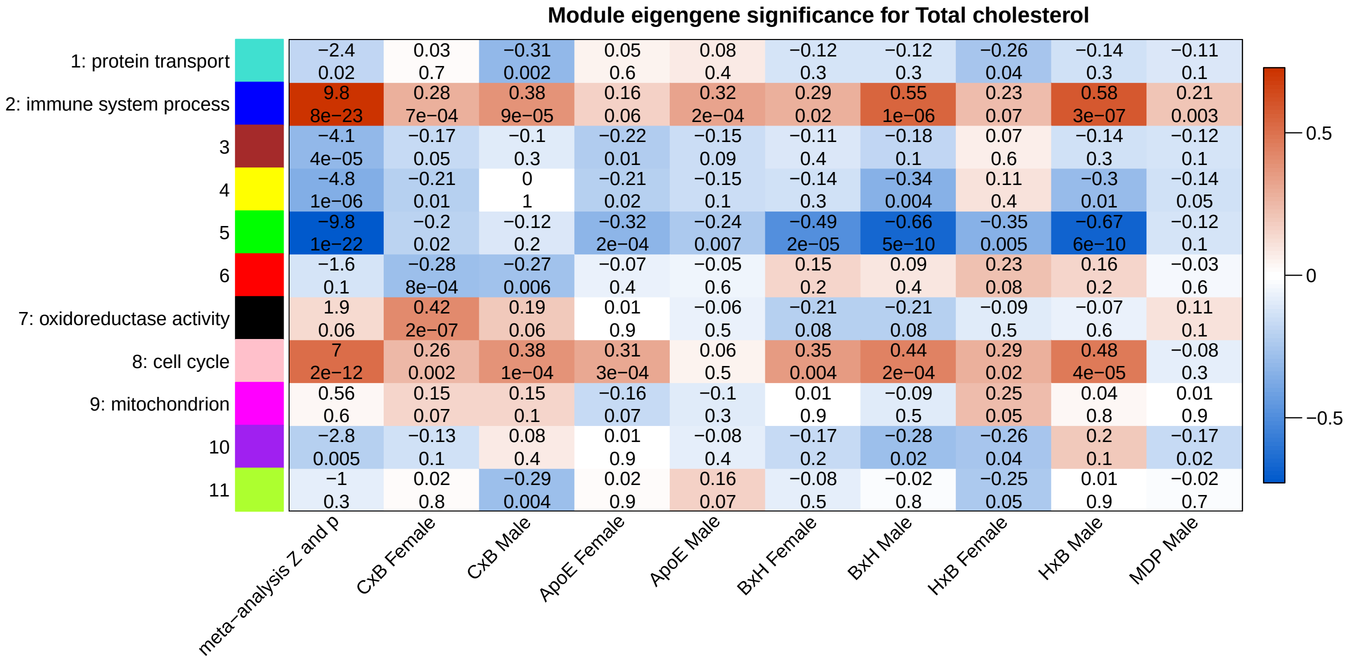


**Figure S8.** Consensus eigengene significance for age across 9 mouse liver expression data sets. Each row of this table corresponds to one module identified in the consensus module analysis. Modules are labeled by the module number and (where appropriate) a GO-derived functional label. Columns 2–10 give robust correlations between total cholesterol and the module eigengenes as well as the associated p-values. The first column shows the meta-analysis Z statistic and the corresponding p-value. We observe that several modules attain strong statistical significance in the meta-analysis of the 9 data sets. The most strongly associated module is module 2. The association between age and the module eigengene is consistent in all 9 data sets, lending further credence to the meta-analysis result. Hence, we focus our analysis on this module.

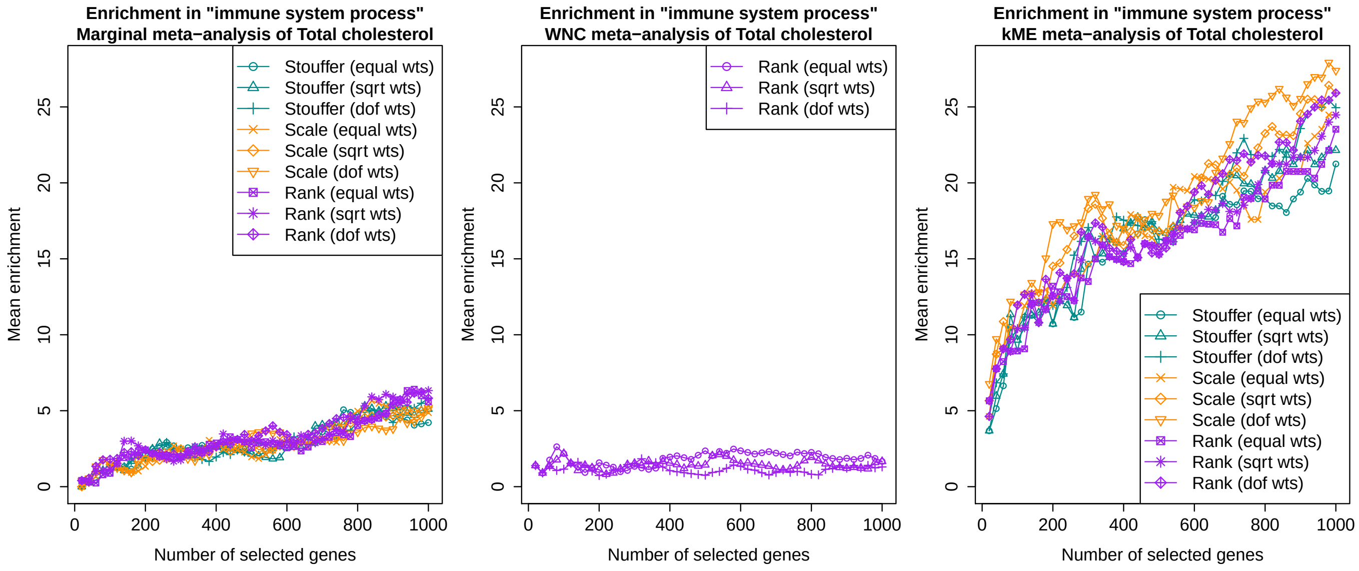


**Figure S9.** Enrichment score (defined as $-log_{10}(p)$ with $p$ being the Bonferroni-corrected enrichment p-value) in in GO term "immune system process" as a function of the number of top selected genes by a variety of meta-analysis (MA) methods applied to **9 mouse liver expression data sets**. The left panel shows enrichment of genes selected by 6 marginal MA methods, while the right panel shows the analogous enrichment of genes selected by MA of module membership in consensus modules. Meta-analysis of module membership in consensus modules selects genes with much higher enrichment than marginal meta-analysis methods.

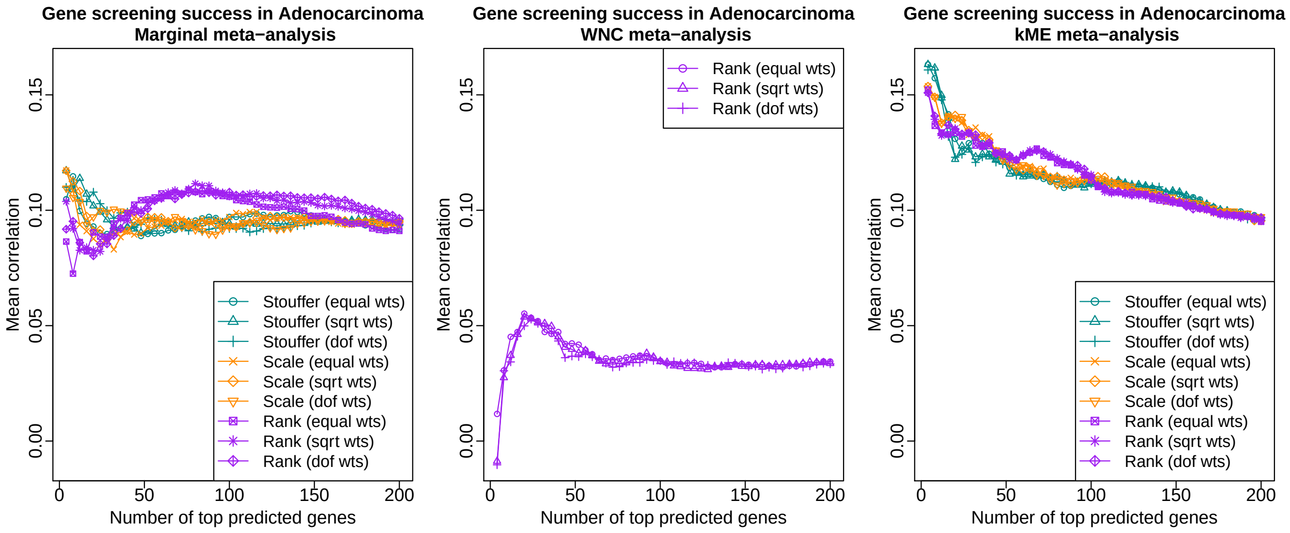


**Figure S10.** Validation success ($y$-axis), measured as the mean correlation of selected genes with survival time deviance in an independent validation data set, as a function of the number of top selected genes ($x$-axis) in the adenocarcinoma expression data (Application 1). In this application, meta-analysis of module membership (right) outperforms marginal meta-analysis (left).

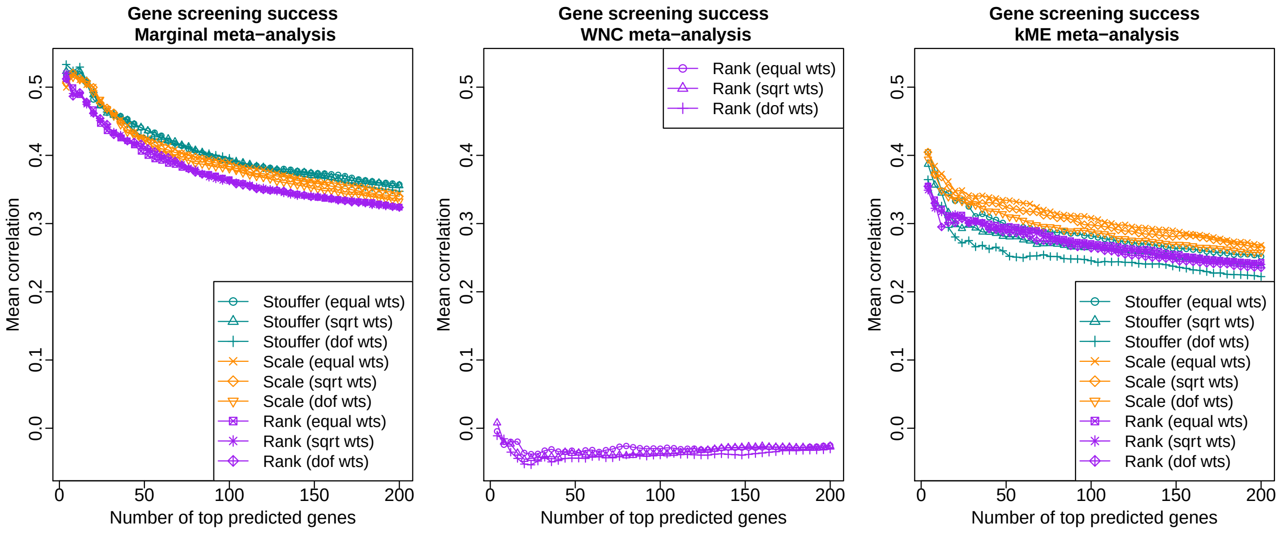


**Figure S11.** Validation success ($y$-axis), measured as the mean correlation of selected genes with survival time deviance in an independent validation data set, as a function of the number of top selected genes ($x$-axis) in the methylation data (Application 2). In this application, marginal meta-analysis (left) outperforms meta-analysis of module membership (right).

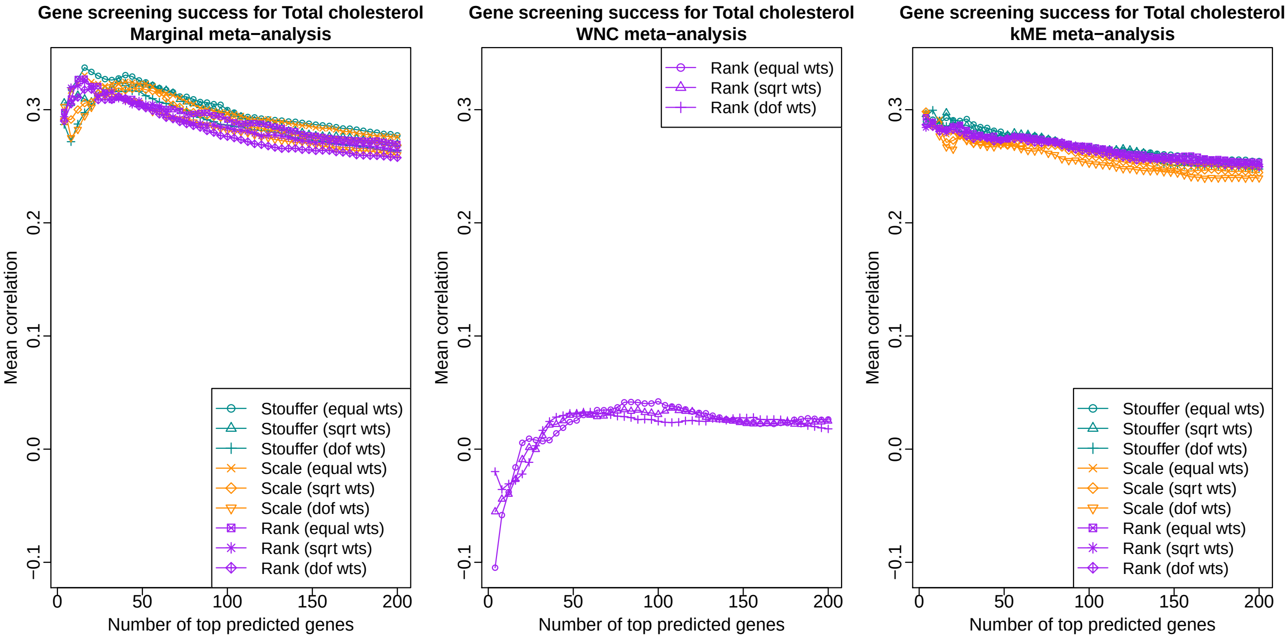


**Figure S12.** Validation success ($y$-axis), measured as the mean correlation of selected genes with survival time deviance in an independent validation data set, as a function of the number of top selected genes ($x$-axis) in the mouse liver expression data (Application 3). In this application, marginal meta-analysis (left) outperforms meta-analysis of module membership (right).

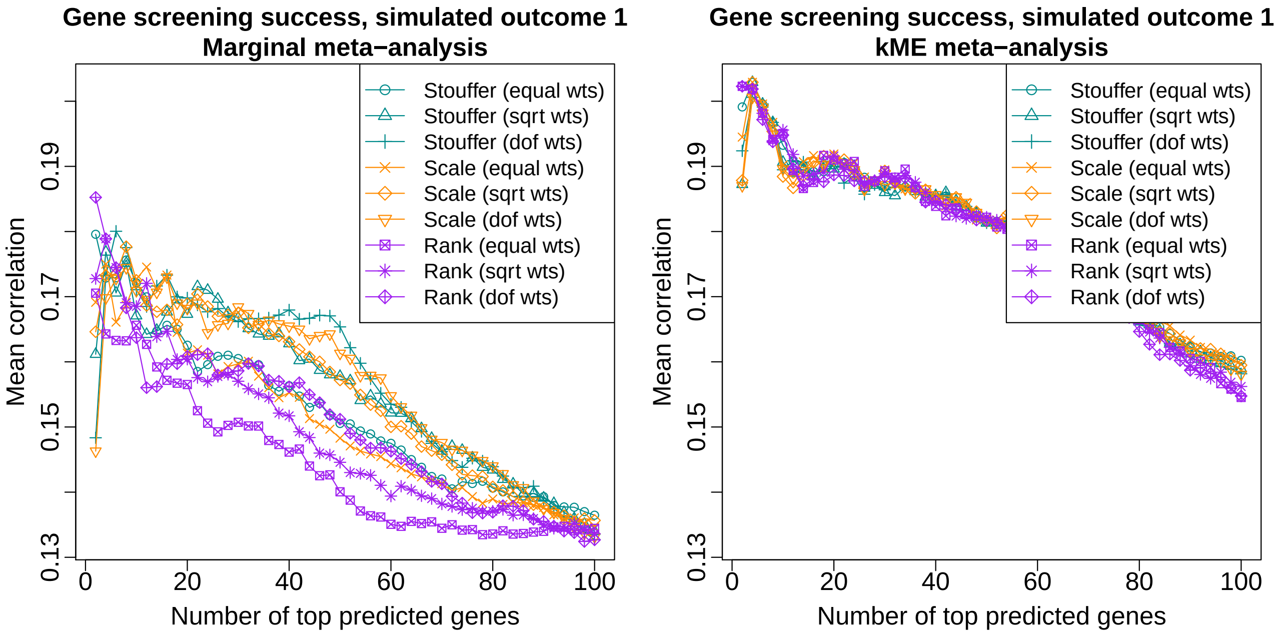


**Figure S13.** Validation success ($y$-axis), measured as the mean correlation of selected genes with clinical trait in an independent validation data set, as a function of the number of top selected genes ($x$-axis), for simulated clinical trait 1. In this simulation, meta-analysis of module membership (right) outperforms marginal meta-analysis (left).

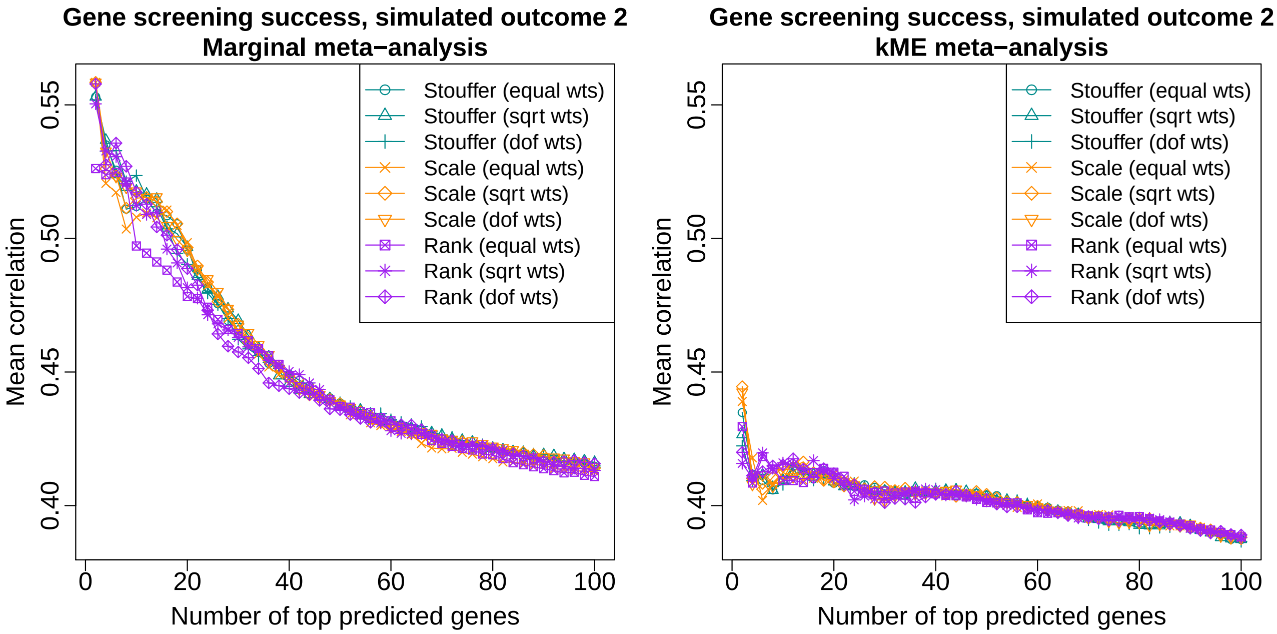


**Figure S14.** Validation success ($y$-axis), measured as the mean correlation of selected genes with clinical trait in an independent validation data set, as a function of the number of top selected genes ($x$-axis), for simulated clinical trait 2. In this simulation, marginal meta-analysis (left) outperforms meta-analysis of module membership (right).
